# Learning PDE to Model Self-Organization of Matter

**DOI:** 10.3390/e24081096

**Published:** 2022-08-09

**Authors:** Eduardo Brandao, Jean-Philippe Colombier, Stefan Duffner, Rémi Emonet, Florence Garrelie, Amaury Habrard, François Jacquenet, Anthony Nakhoul, Marc Sebban

**Affiliations:** 1 Laboratoire Hubert Curien UMR5516, UJM-Saint-Etienne, CNRS, IOGS, Université de Lyon, F-42023 St-Etienne, France; 2 CNRS, INSA-Lyon, LIRIS, UMR5205, Université de Lyon, F-69621 Villeurbanne, France

**Keywords:** machine learning, PDE solving, neural networks, deep learning, physical knowledge incorporation, self-organization process

## Abstract

A self-organization hydrodynamic process has recently been proposed to partially explain the formation of femtosecond laser-induced nanopatterns on Nickel, which have important applications in optics, microbiology, medicine, etc. Exploring laser pattern space is difficult, however, which simultaneously (i) motivates using machine learning (ML) to search for novel patterns and (ii) hinders it, because of the few data available from costly and time-consuming experiments. In this paper, we use ML to predict novel patterns by integrating partial physical knowledge in the form of the Swift-Hohenberg (SH) partial differential equation (PDE). To do so, we propose a framework to learn with few data, in the absence of initial conditions, by benefiting from background knowledge in the form of a PDE solver. We show that in the case of a self-organization process, a feature mapping exists in which initial conditions can safely be ignored and patterns can be described in terms of PDE parameters alone, which drastically simplifies the problem. In order to apply this framework, we develop a second-order pseudospectral solver of the SH equation which offers a good compromise between accuracy and speed. Our method allows us to predict new nanopatterns in good agreement with experimental data. Moreover, we show that pattern features are related, which imposes constraints on novel pattern design, and suggest an efficient procedure of acquiring experimental data iteratively to improve the generalization of the learned model. It also allows us to identify the limitations of the SH equation as a partial model and suggests an improvement to the physical model itself.

## 1. Introduction

Self-organization is prevalent in Nature. It is responsible for the interesting patterns and structures that we observe in systems outside equilibria, from the clouds of Jupiter to how the leopard got his spots. Without self-organization, we would observe mostly disordered states with no discernible structure [1].

Laser-irradiated surfaces are a chief example of a self-organizing system, as one observes coherent, aligned, chaotic, and complex patterns that emerge at the microscale and the nanoscale [2]. These patterns are of great practical interest, with a number of potentially groundbreaking optical, hydrophobic and microbiological applications suggested in the literature [3,4,5,6]. Laser texturing can be used as a method to reduce bacterial colonization of dental and orthopedic implants [7], as it can change surface wettability [8,9] and morphology, the interplay of which affect antibacterial properties [10] of the surface.

Laser-induced periodic structures on stainless steel surfaces were also shown to act as a surface grating that diffracts light efficiently [11,12,13]. Since the orientation of the induced structures depends strongly on laser parameters, and it is possible to have multiple orientation structures with spatial overlap, surfaces can be decorated in such a way that different colors and patterns appear when white light is irradiated on the surface from different directions or even selectively displayed using structural color, with applications to encryption and anti-counterfeiting.

Crucially, laser texturing is reliably reproducible, meaning that these groundbreaking applications have the potential to be turned into industrial processes. In this respect, laser texturing has significant advantages over other surface functionalization methods such as electrochemical etching [14], for example, laser-induced structures can be generated in a simple, single-step process [5] that is reliable, reproducible and scalable, applicable to a great variety of materials [15], and large surfaces [16].

Put simply, under controlled experimental conditions, observed patterns are a function of the laser parameters. This functional dependence is complex, with abrupt boundaries between pattern types for continuous variation of laser properties [17]. Pattern features of interest, such as rotational invariance, characteristic length, and feature height, for example, also show interesting dependence on laser parameters [2,17,18] and novel structures that can be reproduced reliably have recently been observed [18,19].

The variety of patterns and their potentially groundbreaking applications, combined with the controllability and reproducibility of laser-induced structure formation, motivate an exhaustive search of laser parameter space. This search is unfortunately impractical, as each experimental manipulation is costly and time-consuming. Having a model to guide the initialization of laser parameters would thus be of great interest.

A first solution to guide laser parameter selection would be to have a physical model explicitly relating pattern characteristics and laser parameters. Such a model is however not available. While a physical process has recently been proposed to explain the hexagonal patterns observed in experiments [17], the full picture is complex and strict conditions are required for the process to occur upon ultrashort laser irradiation [2]. The photoexcited material evolves in a non-deterministic way due to stochastic surface roughness that may trigger local nonlinear optical response and collective thermomechanical response. In that far from equilibrium conditions, a deterministic approach is able to explain specific nanostructuring regimes [19] but fails to predict the coexistence and the transition between several nanopatterns.

In the absence of an explicit physical model, a second solution to guide laser parameter selection is to use machine learning, which can produce data-driven models with remarkable results. The quality of these results, however, depends on the quantity of training data available. More precisely, drawing from the language of statistical learning theory [20], let *h* be some hypothesis (a model) chosen from some class of hypotheses H of a given complexity *C* (such as Vapnik-Chervonenkis dimension, Rademacher complexity, uniform stability or algorithmic robustness to cite a few), which is chosen by using some algorithm based on minimizing the expected loss (intuitively, expected error) on training data $X_{train}$ drawn from an unknown distribution *D*. Then ΔE, the expected difference in error that we make by evaluating our model on unseen test data $X_{test}$ drawn from the same distribution is bounded above by *g*, a function of the model complexity and the number of training examples $|X_{train}|$. Crucially, it can be shown that *g* is an increasing function of model complexity *C* and g→0 as $|X_{train}|\to \infty$

(1) $$\begin{array}{c} \Delta E\leq g(C,|X_{train}|), \end{array}$$

which means that to keep ΔE small with little data, we must keep the model complexity small. In this sense, the complexity of the model is bounded by the quantity of data: with little data, we can only learn simple models. This is precisely the case with laser-induced pattern experimental data since the difficulty in data acquisition both motivates and hinders the construction of a machine learning model. This situation is far from being exceptional, as most “natural” scenarios are neither in the high data regime [21], nor in the high model, little data regime [22].

**Figure 1:**
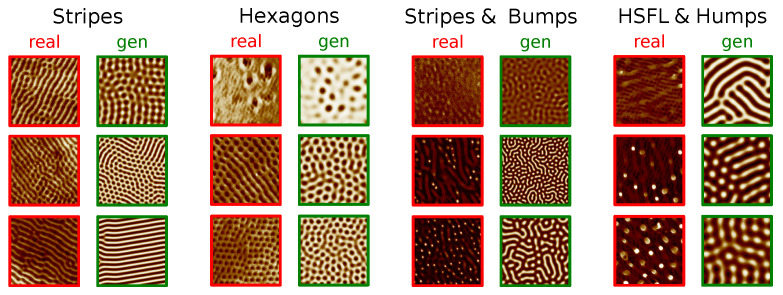
Comparison between real SEM images (**red**) and SH-generated images (**green**). SH-generated images are able to reproduce a variety of patterns (e.g., stripes, hexagons, bumps, HSFL, humps) and scales. A couple of model simplifications can be observed in this comparison: first, since the SH model is an isotropic model, global symmetries are only apparent (e.g., oriented stripes), whereas SEM images retain some measure of global symmetry from laser polarization. Second, since the SH model is a single scale model (in the sense that there is a single critical wavelength), it can only match a single pattern, even for SEM images that contain a superposition of patterns (such as e.g., 7th column, top image).

There are a variety of methods and techniques to learn from data, in this case, namely by integrating physical information to guide the ML model. This collection of techniques is generally grouped under the “Physics-guided” or “Physics-informed” machine learning topic where, among other things, some approaches aim to integrate physical knowledge in the form of a PDE to solve a certain task [23,24,25,26,27]. This paper falls into the scope of this scenario. As a physical model of the convective process that is at the origin of the observed physical structures, we use in this paper the *Swift-Hohenberg* partial differential equation (PDE) on the plane [28], a simple and well-studied model of complex pattern formation under Rayleigh-Bénard convection [1]. We leverage this PDE because, in spite of it being a considerable simplification with respect to the actual process taking place in laser irradiated surfaces, it is still compatible with the physical situation. Pattern-like solutions of the Swift-Hohenberg (SH) equation are remarkably similar to the ones that we can observe in the irradiated surfaces Scanning Electron Microscope (SEM) images, as can be seen in Figure 1. In spite of its simplicity and longevity, the variety of pattern-like solutions of the SH equation still makes it a topic of active research [29].

The main objective of this paper is to design new physics-guided ML techniques that allow us to integrate **partial** physical information and learn with **few data** the relationship between patterns and laser parameters *given that the patterns were produced via a SH convective instability*. While the complexity of the resultant model is still bounded by the same quantity of data, we expect it to be more faithful. To be precise, although the difference in error ΔE in (1) might be small, the minimum error that can be attained in H, which measures the quality of our model, might still be quite large. But if integrating physical knowledge H makes it more compatible with the physical situation (in this sense more faithful), the minimum error will be smaller. As an extreme example, consider learning the sine function, which has an infinite power series expansion (complex) and a one-term expansion in Fourier series (simple).

**Figure 2:**
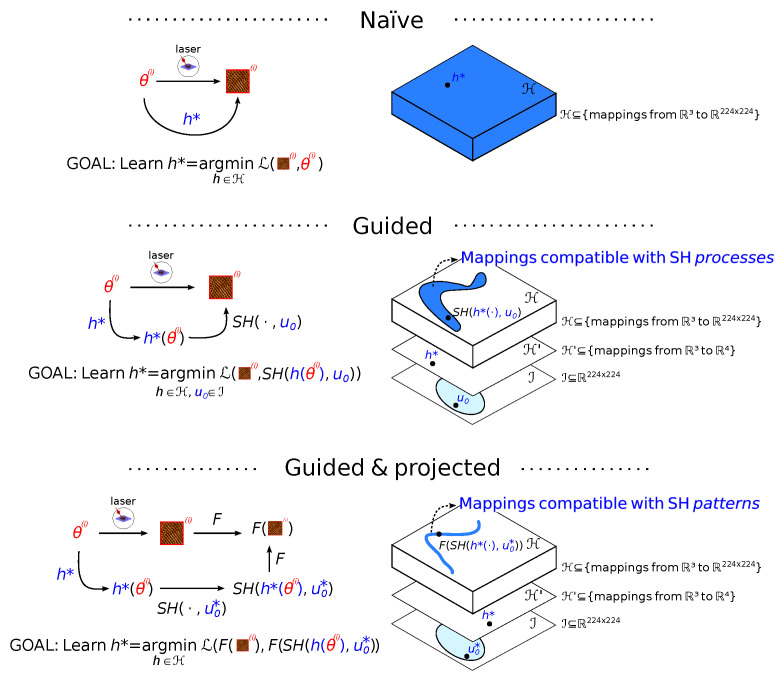
(**top**) The naive approach to learning the relationship between laser parameters $\theta \in R^{3}$ and fields $\in R^{224\times 224}$ (224 by 224 pixels SEM images) is to seek the minimizer $h^{*}$ of the loss L in the large set H. With few observations, this is unfeasible. (**middle**) Physical knowledge can guide the search by restricting it to mappings that are compatible with the SH model—the much smaller subset of H in blue. The learned mapping is now only between $\theta \in R^{3}$ and SH parameters $\in R^{4}$. We still have to minimize over initial conditions $u_{0}\in I$, to which we do not have experimental access. Again this is unfeasible. (**bottom**) Since we are only interested in general pattern features, and the SH model is based on a self-organization process, a feature mapping *F* exists such that in the image of *F*, patterns can be described using model parameters alone. We safely ignore initial conditions (e.g., choosing $u_{0}^{*}$) and seek the minimizer over mappings that are compatible with SH *patterns*—the much smaller subset of H in blue. This is feasible with little data.

In situations where similar patterns are observed, there is considerable information contained in the *parameters* of the Swift Hohenberg equation, which determines the *type* and general features of the pattern solutions for a range of initial conditions. In this sense, for the purpose of predicting novel patterns, there is too much physical information in the SH solutions, and a feature transformation *F* exists such that in the image of *F*, patterns can be effectively described using model parameters alone (see Figure 2 for details).

As we shall see in the sequel, this key insight informs our contribution which is five-fold: **(i)** we present a Bayesian inference formulation for solving the dual inverse problem of estimating state and model parameters in the case of self-organization, **(ii)** we design an efficient solver of SH PDE allowing the generation of a large amount of SH (parameters-patterns) pairs of data **(iii)** from this large dataset, we learn a differentiable-Neural Network (NN) surrogate of the SH PDE, **(iv)** we leverage this pre-trained NN, on the one hand, and the SH (parameters-patterns) pairs, on the other, to learn two alternative end-to-end models from the laser parameters to the patterns obtained by laser irradiation; **(v)** we conduct experiments showing a good agreement between the generated images and experimental data for some parameter ranges, which can serve as a guide for laser initialization and suggest improvements to the SH model.

The rest of this paper is organized as follows: in Section 2 we frame the problem of learning novel laser patterns by integrating partial physical information in the form of a PDE and discuss related work. In Section 3 we present a framework for solving the dual inverse problem with few data by integrating partial physical information in the context of systems with self-organization. In Section 4 we apply this framework to the specific case of learning novel laser-induced patterns on monocrystalline Nickel (Ni) at the nanoscale: we begin by briefly presenting the SH equation as a partial model of the physical situation in Section 4.1.1; we proceed by presenting a pseudo-spectral second-order solver of the SH equation combining accuracy and speed in Section 4.1.2; we continue by discussing, in Section 4.2, a particular feature mapping that we chose for our problem, which allows considerable simplification, and that we validate via a quality measure based on expert clustering results in Section 3.4.1; and finally, we present two alternative models to learn the relationship between laser parameters and SH parameters, which will allow us to predict novel patterns in Section 4.3. In the next Section 5 we present and discuss experimental results; notably, we see that pattern features are correlated and that more than one SH process may be at play. We conclude in Section 6 and discuss future research.

## 2. Related Work

Given a physical phenomenon modeled by a PDE, the problem of predicting the result of measurements is known as the *forward problem*, while estimating unobserved states and parameters that characterize the system—which is needed to solve the forward problem—is called the *inverse problem*. The solution of the forward problem for deterministic PDEs is generally unique, but that of the inverse problem is not. It is rather naturally expressed as a probability distribution, which motivates a rigorous formulation of the inverse problem in terms of Bayesian inference, with well-established methods going back to Laplace [30].

Incorporating prior knowledge and combining it with data is the key problem in Bayesian estimation. Since priors and data are domain-specific, it should come as no surprise that methods to tackle the inverse problem have been developed in parallel in several domains where physical knowledge can be expressed in terms of a PDE and data is abundant.

In geophysics and climate science, physical models are sophisticated and well-established but there is only partial information about state: satellite data, for example, is given in patches, but forecasting using the physical model requires knowledge of the full state; solving this inverse problem in this domain is commonly done using a collection of methods known as *data assimilation* [31].

In the physics community, on the other hand, one can often carefully prepare experiments to set the initial state, and the main goal is now physical model development or validation. When the initial state is known, the inverse problem of finding the distribution of model parameters is thus the main focus. It is known as *model calibration* [32], and a host of recent results exist using machine learning techniques, notably deep learning [33], to learn the parameters of either the full model (which is typically carefully constrained as to incorporate the right biases, and can thus be seen as already incorporating physical knowledge) or a correction to incomplete physical knowledge [34] (an *augmented* model) from data.

More specifically in photonics, the focus is on inverse design [35]—algorithmic techniques for discovering optical structures based on desired functional characteristics —, with neural networks being used to speed up optimization of nano-photonics structures, by replacing the forward model with a much faster to evaluate neural network surrogate [36,37], for example. The task is reminiscent of our own. Unlike in our case, however, the physical model is assumed to be complete, the system state can be prepared, and data is abundant. Crucially, there is a specific functional characteristic to optimize for, whereas we are interested in exploration rather than design.

In reality, there is always some uncertainty with respect to either model parameters or system state. To solve the *joint* inverse problem of finding state and model parameters, several approaches involving machine learning were recently proposed in the climate sciences. The main idea is to alternate a data assimilation step to estimate state and a machine learning step to learn model parameters from data [38,39,40,41]. Because in the climate science case the dynamics are generally well-known, the models that are jointly learned can be seen as *corrections* to partial physical knowledge given as a governing PDE, and the goal is to learn them from data (although the correction itself also commonly incorporates physical knowledge or symmetries in the form of constraints imposed during training [42,43,44,45,46] and/or in the network architecture [38,47]).

In these terms our task of predicting new laser patterns can be framed as follows: we have a complex system in which the laser-matter interaction takes place; $X_{t}$, the state of this system at time *t* is unknown but for its noisy image through an observation operator $Y_{t}=HX_{t}+\epsilon$, which consists in the laser parameters and the SEM images of the material at the zone of incidence of the laser spot after solidification (the solidification time is not fixed and depends, among other things, on laser fluence). As in the climate science approaches above, we have an explicit partial model for the transition from state $X_{t}$ to $X_{t+1}$, the Swift-Hohenberg equation with unknown parameters φ, which we can see as a first order model of the physical phenomenon, and we have no access to the hidden state. Crucially, unlike these approaches, we have a single observation for each evolution and several orders of magnitude fewer evolution data. This makes the dual inverse problem of inferring initial state and model parameters unfeasible, and the aforementioned methods inapplicable.

We shall overcome this difficulty by recasting the problem in such a way that for our purposes, in the case of self-organization pattern forming dynamics, the state determination inverse problem is unnecessary, in the sense that a feature transformation *F* exists such that in the image of *F*, patterns can be effectively described via model parameters alone. This feature transformation is shown to exist in Section 3.4.2 for the case of models with pattern-like solutions (which is our case, as detailed below in Section 4.1.1). This turns the high-dimensional ill-posed inverse problem into a much lower-dimensional problem of learning the relationship between laser parameters and model parameters using data.

## 3. Integrating Partial Physical Information to Learn with Few Data and No Knowledge of Initial Conditions

In this section, we derive a principled approach to the problem of learning a complex relationship between an observable quantity θ and a physical field *u*, given only little data, by taking advantage of partial contextual physical knowledge in the form of a differential equation on *u* not explicitly depending on θ, in the absence of knowledge of initial conditions. We strive for generality but we shall refer to the main subject of this paper for examples and clarification.

This problem is fundamentally difficult in three interacting ways: *first*, the dearth of experimental data makes learning a complex relationship directly, from data alone, impossible. This is usually where a physical model can be of assistance, typically via data augmentation: one uses a differential equation solver—or a neural network surrogate of a differential equation solver—to generate more θ,u pairs in a principled, physically consistent manner, which we can then use to complement the small dataset size. Even if the physical information is not complete, it can still be of use: if the model is only approximate, one splits the field into two components, one which is exactly modeled by the physical model, and a second which can be seen as a perturbation of the former, and which can be learned from data [34]. But producing a model explicit on θ is the *second* difficulty, particularly in the earlier stages of the experimental process or in the case where θ are difficult to interpret in terms of known physical quantities commonly used to parameterize the physical model. This is not an insurmountable difficulty: we may lack explicit physical knowledge, but it is rarely the case that we approach a physical situation without any sort of background knowledge. In most situations, a *general* model relating common physical observables and the physical field can be produced, derived from first principles or symmetry arguments. This can be a considerable simplification, as it may allow us to turn the problem of learning the relationship between the θ and *u* into one of learning the relationship between the θ and the general model parameters φ. As the dimension of φ is generally lower than the dimension of *u*, it is much easier to learn the latter. Herein lies the *third* difficulty: if the general model is good and the observables are sufficiently informative, one can hope that the number of data required for calibration be small. But calibration data is of a different nature as it is typically done via a series of carefully designed experiments [1]. Learning the parameters of a differential equation in time typically involves knowledge of *u* at different times, which is not necessarily part of the original θ,u data—we need then knowledge of initial conditions for *u*.

We propose to address these difficulties as follows: we first show that in the case of self-organization, and pattern-forming processes, the information contained in the initial conditions $u_{0}$ is *small*, in the sense that a non-injective feature transformation *F* exists such that they can be losslessly expressed in the lower-dimensional $F(u_{0})$, which makes the dependency on initial conditions less important. We then proceed by making two assumptions on the physical process and its relationship with the observables, allowing us to propose a method to take advantage of self-organization physical knowledge to learn with little data. We note that these assumptions hold in a number of interesting cases, namely climate models, where our method could be also applied to find the relationship between observed quantities and features of rare events, for example (extreme event prediction, of which, by definition, we have few data).

### 3.1. Problem Statement

**Figure 3:**
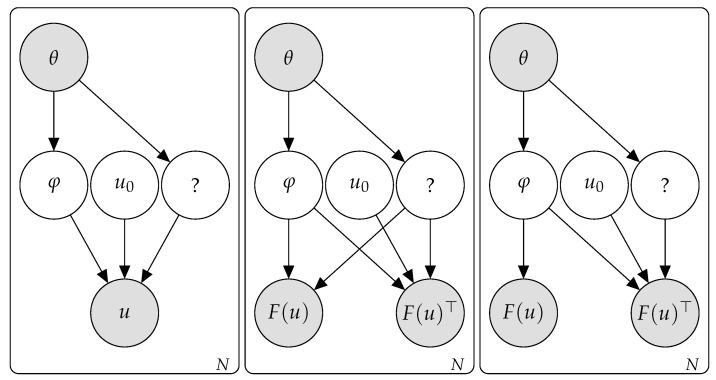
(**Left**): Observable θ influences a physical process φ, among others (marked “?”), which, together with unobserved initial conditions $u_{0}$, determine observed field *u*. (**Center**): A feature transformation exists such that $u=(F\left(u\right),{F(u)}^{⊥})$, and the observable F(u) is useful and independent of initial conditions $u_{0}$. (**Right**): in addition, the feature transformed observable is not affected by the other physical processes.

Consider a physical field u(x,t):=u which we believe is mainly the result of a certain physical process, the evolution of which can be described in terms of physical process parameters φ and initial conditions $u_{0}$ which are compatible with the physical situation and by a PDE $\dot{u}=f\left(\varphi ,u_{0}\right)$ (throughout this section we assume periodic boundary conditions and that *u* is a real field; we further assume that conditions are satisfied such that *u* is unique; existence is posited since we are assuming that the process is modeled by this equation and we observe the fields). Assume we have no knowledge of either φ or $u_{0}$—although the latter is assumed to belong to a large set $U_{0}$ of “*reasonable*” initial conditions compatible with the physical situation. We also assume that a certain *observed* quantity θ (which in our case will correspond to the laser parameters) exists which affects φ—although it may also affect other unknown latent variables, which may, in turn, affect *u* (Figure 3, left).

We would like to sample from the distribution of *u* given a certain value of the observed quantity θ. Unfortunately, since we have no knowledge of the initial conditions, p(u|θ) cannot be calculated directly

$$\begin{array}{cc} p(u|\theta ) & =\sum_{u_{0}}\sum_{\varphi }p\left(u,u_{0},\varphi |\theta \right)\\ \; & =\sum_{u_{0}}\sum_{\varphi }p\left(u|u_{0},\theta ,\varphi \right)p\left(u_{0},\varphi |\theta \right), \end{array}$$

(where sums are to be replaced by integrals in the continuous case) and we shall need to make a hypothesis about the physical process to proceed.

### 3.2. Two Hypotheses on the Physical Process

The distribution p(u|θ) may be impossible to calculate directly, but the quantity of interest is often not *u*, but rather a certain function of *u*, F(u), where F(u) is typically simpler than *u*, which motivates the following hypotheses:

**Hyphothesis** **1.** *A function F of the field u exists such that the knowledge of the physical process φ suffices to determine the likelihood of F(u) conditioned on θ. Precisely, $p\left(F\left(u\right)|u_{0},\theta ,\varphi \right)=p\left(F\left(u\right)|\theta ,\varphi \right)$. We call* feature space *the image of F on the domain of u.*

Under Hypothesis 1, and assuming that the initial conditions are independent of the physical process φ and the observed quantity θ (as shown in Figure 3), we have

$$\begin{array}{cc} p(F(u)|\theta ) & =\sum_{u_{0}}\sum_{\varphi }p\left(F\left(u\right)|u_{0},\theta ,\varphi \right)p\left(u_{0},\varphi |\theta \right)\\ \; & =\sum_{u_{0}}\sum_{\varphi }p\left(F\left(u\right)|\theta ,\varphi \right)p\left(u_{0}|\varphi ,\theta \right)p\left(\varphi |\theta \right)\\ \; & =\sum_{u_{0}}\sum_{\varphi }p\left(F\left(u\right)|\theta ,\varphi \right)p\left(u_{0}\right)p\left(\varphi |\theta \right)\\ \; & =\sum_{\varphi }p\left(F\left(u\right)|\theta ,\varphi \right)p\left(\varphi |\theta \right). \end{array}$$

To proceed, we make the following additional assumption:

**Hyphothesis** **2.** *The observed quantity θ determines the physical process: p(φ|θ) is peaky, in the sense that there is a $\tilde{\varphi }$ such that p(φ|θ)=0 for $\varphi \neq \tilde{\varphi }$. In particular, if p is continuous, $p\left(\varphi |\theta \right)=\delta (\varphi -\tilde{\varphi }\left(\theta \right))$, where δ(·) refers to the peaky distribution related to physical process.*

Under this assumption, $\tilde{\varphi }$ is a function of θ, which implies that θ is at least locally a function of $\tilde{\varphi }$ and we can write

$$\begin{array}{cc} p(F(u)|\theta ) & =\sum_{\varphi }p\left(F\left(u\right)|\theta ,\varphi \right)p\left(\varphi |\theta \right)\\ \; & =p\left(F\left(u\right)|\theta \left(\tilde{\varphi }\right),\tilde{\varphi }\right)p\left(\tilde{\varphi }|\theta \right)\\ \; & =p(F\left(u\right)|\theta \left(\tilde{\varphi }\right),\tilde{\varphi })\\ \; & =p(F\left(u\right)|\tilde{\varphi }). \end{array}$$

How can we find $\tilde{\varphi }\left(\theta \right)$?

### 3.3. Learning by Maximizing the Likelihood

Our general method to learn $\tilde{\varphi }\left(\theta \right)$ is to maximize the likelihood of the observations. In the sequel, we propose two strategies to do so: one in which we maximize it directly, and another in which we maximize a lower bound.

#### 3.3.1. Maximizing the Likelihood Directly

Having access to experimental data ${\left\{\theta^{i},u^{i}\right\}}_{i=1\ldots N}$, we can parameterize $\tilde{\varphi }$ with a Neural Network (α), for example, and maximize the log likelihood of observing the $F(u^{i})$:

(2) $$\begin{array}{c} \bar{\alpha }=arg max\sum_{i=1}^{N}\log p\left(F\left(u^{i}\right)|\tilde{\varphi }\left(\theta^{i}\right);\alpha \right). \end{array}$$

If *u* is high-dimensional, the relationship between F(u) and $\tilde{\varphi }$ is potentially complex, and thus requires *N* large to model satisfactorily. Having physical knowledge in the form of a differential equation solver $u=Solver(\tilde{\varphi },u_{0})$, however, considerably simplifies the problem.

In feature space, for a process respecting Hypothesis 1, we can choose arbitrary initial conditions and fit only the relationship between θ and the *parameters* of the differential equation, which generally has a much lower dimension than the field *u*. Since this is a much simpler task, we expect that a much smaller *N* will suffice to produce a satisfactory model.

Assuming data is generated i.i.d. from a fixed-variance Gaussian distribution, this corresponds to minimizing the mean squared error between the images, through *F*, of experimental fields, and fields generated by the PDE solver for an arbitrary initial condition $u_{0}\in U_{0}$.

(3) $$\begin{array}{c} \bar{\alpha }=arg min\frac{1}{N}\sum_{i=1}^{N}{∥F\left(u^{i}\right)-F\left(Solver\left({\tilde{\varphi }}_{\alpha }\left(\theta^{i}\right),u_{0}\right)\right)∥}^{2} \end{array}$$

Note that this optimization is more conveniently done with a differentiable *surrogate* of the solver.

#### 3.3.2. Maximizing a Lower Bound of the Likelihood

Note that

$$\begin{array}{cc} \log\left(p\left(F\left(u\right)|\theta \left(\tilde{\varphi }\right),\tilde{\varphi }\right)p\left(\tilde{\varphi }|\theta \right)\right) & =\log p\left(F\left(u\right)|\theta \left(\tilde{\varphi }\right),\tilde{\varphi }\right)+\log p\left(\tilde{\varphi }|\theta \right)\\ \; & =\log p\left(F\left(u\right)|\tilde{\varphi }\right)+\log p\left(\tilde{\varphi }|\theta \right). \end{array}$$

(we recall that even though we are under the conditions of Hypothesis 2—φ is a function of θ —, we don’t know which function that is, which explains keeping around the term $\log p(\tilde{\varphi }|\theta )$, which we will maximize for experimental data in order to find $\tilde{\varphi }$).

Let ${\tilde{\varphi }}_{1}$ be the maximizer of the first term, ${\tilde{\varphi }}_{1}={arg max}_{\tilde{\varphi }}\log p\left(F\left(u\right)|\tilde{\varphi }\right)$. Then

(4) $$\begin{array}{c} \max_{\tilde{\varphi }}\left\{\log p\left(F\left(u\right)|\tilde{\varphi }\right)+\log p\left(\tilde{\varphi }|\theta \right)\right\}\geq \log p\left(F\left(u\right)|{\tilde{\varphi }}_{1}\right)+\log p\left({\tilde{\varphi }}_{1}|\theta \right). \end{array}$$

Provided we find ${\tilde{\varphi }}_{1}^{i}$ for each $u^{i}$, we can replace the log-likelihood maximization objective with that of maximizing a lower bound:

(5) $$\begin{array}{c} \bar{\alpha }=\underset{\alpha }{arg max}\sum_{i=1}^{N}\log p\left({\tilde{\varphi }}^{i}|\theta^{i};\alpha \right) \end{array}$$

which, repeating the argument above, corresponds to minimizing the mean squared error,

$$\begin{array}{c} \bar{\alpha }=\underset{\alpha }{arg min}\frac{1}{N}\sum_{i=1}^{N}{∥{\tilde{\varphi }}^{i}-{\tilde{\varphi }}_{\alpha }\left(\theta^{i}\right)∥}^{2}. \end{array}$$

We can replace this task with maximizing a lower bound, *after* having found the first maximizer. It remains to find the ${\tilde{\varphi }}^{i}$. To do so, we note that by having an efficient solver and assuming sufficient regularity, one can pre-generate sufficiently many fields $U_{g}={\left\{u_{g}^{k}\right\}}_{k=1\ldots M}$ such that the expected distance to the nearest neighbor in feature space, given by *F*, is as small as one would like:

$$\begin{array}{cc} \delta & =\frac{1}{M}\sum_{k=1}^{M}\min_{j\neq i}{∥F\left(u_{g}^{k}\right)-F\left(u_{g}^{j}\right)∥}^{2}\\ \; & =\frac{1}{M}\sum_{k=1}^{M}{∥F\left(u_{g}^{k}\right)-F\left({\hat{u}}_{g}^{k}\right)∥}^{2}, \end{array}$$

where we denoted ${\hat{u}}_{g}^{k}$ as the nearest neighbor in $U_{g}$ of $u_{g}^{k}$, in feature space. Denoting $Solver\left({\tilde{\varphi }}_{\alpha },u_{0}\right):=u^{\alpha }$ for simplicity and ${\hat{u}}^{\alpha }$ its nearest neighbor in $U_{g}$, we have

$$\begin{array}{cc} \min_{\alpha }\frac{1}{N}\sum_{i=1}^{N}{∥F\left(u^{i}\right)-F\left(u^{\alpha }\right)∥}^{2} & =\frac{1}{N}\sum_{i=1}^{N}\min_{\alpha }{∥F\left(u^{i}\right)-F\left({\hat{u}}_{p}^{i}\right)+F\left({\hat{u}}_{p}^{i}\right)-F\left(u^{\alpha }\right)∥}^{2}\\ \; & \leq \frac{1}{N}\sum_{i=1}^{N}{∥F\left(u^{i}\right)-F\left({\hat{u}}_{p}^{i}\right)∥}^{2}+\min_{\alpha }\frac{1}{N}\sum_{i=1}^{N}{∥F\left({\hat{u}}_{p}^{i}\right)-F\left(u^{\alpha }\right)∥}^{2}\\ \; & \leq \frac{1}{N}\sum_{i=1}^{N}{∥F\left(u^{i}\right)-F\left({\hat{u}}_{p}^{i}\right)∥}^{2}+\delta \\ \; & \leq 2\delta . \end{array}$$

Since δ→0 we can set ${\tilde{\varphi }}^{i}$ as the solver parameter of the nearest neighbor in feature space, among pre-generated fields, of $u^{i}$.

### 3.4. Generality of the Two Hypotheses

The hypotheses above correspond to the following desiderata:

- A useful, simplifying, and separating feature space *F* exists.
- In *F*, the task is independent of initial conditions.
- In *F*, the physical process φ is essentially a function of the observed θ.

which we now examine in turn.

#### 3.4.1. Choosing a Useful, Simplifying, Separating Feature Space

**Figure 4:**
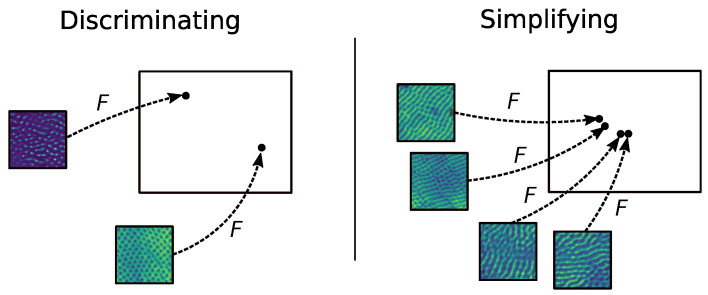
One would like the feature transformation of choice *F* to be simplifying in the sense that it abstracts away pattern minutia while still being discriminating in the sense that patterns which are different will be mapped to different points.

In the exploratory stages of research, an explicit measure of usefulness is typically not available, because future applications are unknown. It is thus important to keep the feature transformation *F* as discriminating as possible. Without imposing further constraints, this is trivially satisfied by choosing a bijection. On the other hand, we want *F* to be simplified, in the sense that it is invariant to quantities that we do not care for. Without further constraints, this objective is satisfied trivially by choosing *F* as a projection to a single point. These two objectives, which we illustrate in Figure 4, are in contradiction, and fulfilling them simultaneously is not trivial.

This strategy could still be pursued in principle by training a model for a set of broadly defined constraints, but doing so is expensive in terms of time and data, and cannot be justified in practice in the early stages of research. The problem is reminiscent of that in [48], in which *F* is called a *physical rescaling*; as in our paper, the assumption is that it is inconvenient to train a model to find such a rescaling, which is thus obtained based on physical knowledge and/or statistical properties leaving the target variables invariant. This method is interesting but, in a setting of partial physical information and little data, explicitly defining discriminating invariant feature transformations based on physical or statistical arguments is challenging.

A reasonable alternative in this setting is to choose a simplifying feature transformation *F* which, applied to similar data, is known to allow a broad task to be performed, which is the strategy that we propose. This has the inconvenience of the feature transformation *F* not being uniquely defined, which is why we now propose a method to compare several such transformations.

For concreteness and the sake of clarity, we present this strategy in the context of our specific task of finding a feature space for predicting novel laser-induced nanoscale patterns (shape and scale). The shape of the patterns observed in SEM images is described by the experts in somewhat loose terms such as `labyrinthine’, `hexagons’, `bumps’, `peaks’. Different patterns are believed to have different applications with different utilities. However, this strategy can in principle be generally used in the context of the early stages of the experimental process, with little data and partial knowledge, and where a specific measure of utility is not yet established.

##### A Quality Measure for the Feature Transformation

We *define* as a measure of the quality of a feature transformation *F* with respect to physically relevant patterns, the accuracy of the classification task where the patterns are classified as the modal pattern in the clusters obtained by k-means in feature space. To see why, consider $u_{i}^{\left(p_{1}\right)},u_{j}^{\left(p_{1}\right)}$ two fields with the same pattern. If *F* is *invariant* with respect to this pattern, then $F\left(u_{i}^{\left(p_{1}\right)}\right)=F\left(u_{j}^{\left(p_{1}\right)}\right)$. If *F* is *discriminating*, for all patterns $p_{k}$, if k≠l then for all i,j, $F\left(u_{i}^{\left(p_{k}\right)}\right)\neq F\left(u_{j}^{\left(p_{l}\right)}\right)$. It follows that if *F* is invariant and discriminating, it solves the task of clustering fields according to patterns. This motivates our definition.

In practice, as mentioned in [48], a fully invariant non-trivial mapping is difficult or impossible to find, which leads us to define invariance with a given tolerance. This motivates our measure of invariance as the accuracy of a distance-based clustering algorithm, which we take as the k-means modal classifier for simplicity.

We note that identification of the modal pattern and choice of the number of clusters *k* require physical knowledge, and were therefore performed by an expert. On the other hand, this knowledge does not have to be explicitly defined, which allows our method to be applied in the context of partial physical knowledge, in the early stages of the research process. Finally, we also note that the choice of invariant, discriminating feature *F* transformation is not unique: if *F* is such a transformation, we can obtain another one by adding a constant feature, for example. All things being equal, we would rather choose an *F* as simple as possible.

#### 3.4.2. Independence of Initial Conditions

In general, we cannot guarantee that a feature transformation exists that allows abstracting away initial conditions, but we now show that this is the case for pattern-forming systems via a deterministic process. To ground intuition, we recall that patterns are often formed because Fourier modes are selectively amplified/attenuated during the dynamics, the set of frequencies that are *not* driven to zero laying on a band around some critical frequency (of which there could be more than one). To see that necessarily implies that the initial conditions are redundant, we begin by establishing notation and a few definitions: Let *u* be a real field defined on a finite interval of length *L* with periodic boundary conditions (the extension to polytopes is straightforward). Setting L=2π for simplicity, in the frequency domain, the maximum wave number compatible with such boundary conditions is 2π/L=1. The possible wave numbers are thus $\frac{2\pi }{L}n=n$ for integers n≥1, and we can write the field as $u=\sum_{n=1}^{\infty }a_{n}f_{n}$ where $f_{n}$ is the Fourier mode with frequency *n* and $a_{n}\in C$ its amplitude.

To each field, we can associate a distribution of its amplitude among the modes by setting $p_{n}=\frac{{\left|a_{n}\right|}^{2}}{\sum_{n=1}^{\infty }{\left|a_{n}\right|}^{2}}$.

Finally, to this distribution we can associate the following Shannon Entropy H(u):

(6) $$\begin{array}{c} H\left(u\right)=-\sum_{n=1}^{\infty }p_{n}\log p_{n} \end{array}$$

the general idea of the proof is the following:

1. Let *u* be a real field on a bounded domain Ω at a fixed time *t*, which is the result of a deterministic physical process for given initial conditions *i*. If that is the case, then there is a function $L_{t}:\Omega \to \Omega$ such that $u=L_{t}\left(i\right)$. The mutual information between the field *u* at time *t* and its initial value *i* is thus I(u,i)=H(u)−H(u|i)=H(u), since $u=L_{t}\left(i\right)$ and $H(L_{t}\left(i\right)|i)=0$.
2. We prove that for general initial conditions the entropy for a self-organization physical process is decreasing H(u)<H(i).
3. If $L_{t}$ were a bijection, an inverse $L_{t}^{-1}$ would exist and $I\left(u,i\right)=I(L_{t}^{-1}\left(i\right),i)=H\left(i\right)$ by the same argument as above, which would imply H(i)=H(u). Since this is not the case, $L_{t}$ must map to a lower-dimensional space.
4. In this lower dimensional space, the initial conditions completely determine the field *u* (identically, since $u=L_{t}\left(i\right)$).
5. Hence, for such a physical process, there is necessarily redundant information in the specification of initial conditions.

It remains to show the following proposition:

**Proposition** **1.** *The entropy of a self-organization process is decreasing for general initial conditions.*

**Proof.**  Consider now a variation in the distribution of amplitudes $\left({\dot{p}}_{1},\cdots ,{\dot{p}}_{k},\cdots \right):=\dot{p}$, where the dot denotes a time derivative. The corresponding time derivative of the entropy is then:

(7) $$\begin{array}{cc} \dot{H} & =-\sum_{n=1}^{\infty }{\dot{p}}_{n}\left(\log p_{n}+1\right) \end{array}$$

(8) $$\begin{array}{cc} \; & :=-\dot{p}\cdot \left(\log p+1\right), \end{array}$$

The maximal change in entropy will be for $\dot{p}$ aligned with logp+1, which is clearly not generally the case for some initial distribution of amplitudes p. For self-organization physical processes, patterns are often formed because a certain range of nodes in the neighborhood of a critical wave number $n_{0}$ is amplified, all others being attenuated. One can generally describe this evolution in terms of the amplitude of each node:

(9) $$\begin{array}{c} a_{n}\left(t\right)=e^{-{\left(n-n_{0}\right)}^{2}t}a_{n}\left(0\right) \end{array}$$

where we dropped the absolute value for notation simplicity (the previous statements are strictly true for a Type 1S instability, near onset, and for a small perturbation of the u=0 solution in the linear approximation, see [1] for details). Assuming that there is no large power mode (all $p_{k}<1/2$, which is indeed the case for k>2 for the maxent distribution of Fourier power spectrum, which is the uniform distribution), all components of the vector logp+1 are negative. Further assuming that the physical process has the property that the $l_{2}$ norm of the field is constant, $\sum_{n=1}^{\infty }|a_{n}(t{)|}^{2}:=1/\alpha$ we have, by replacing (9) in the time derivative of the entropy above, that the entropy of the self-organization process $H_{so}$ decreases for every initial distribution of amplitudes:

$$\begin{array}{cc} {\dot{H}}_{so} & =-\sum_{n=1}^{\infty }{\dot{p}}_{n}\left(\log p_{n}+1\right)\\ \; & =-\alpha \sum_{n=1}^{\infty }\dot{\left(a_{n}^{2}\right)}\left(\log p_{n}+1\right)\\ \; & =-2\alpha \sum_{n=1}^{\infty }{\dot{a}}_{n}a_{n}\left(\log p_{n}+1\right)\\ \; & =\alpha \sum_{n=1}^{\infty }{\left(n-n_{0}\right)}^{2}a_{n}^{2}\left(\log p_{n}+1\right)<0, \end{array}$$

which proves the claim. As we mentioned above, this decrease is not maximal as it is not necessarily so that to low power modes there will correspond a greater decrease in amplitude. □

#### 3.4.3. The Likelihood Is Peaky: φ Is a Function of θ

We cannot control whether or not the likelihood p(φ|θ) is peaky. If it is not, then there are several φ that can be associated with the same θ and the log-likelihood is

$$\begin{array}{cc} \log p(F(u)|\theta ) & =\log\sum_{\varphi }p\left(F\left(u\right)|\theta ,\varphi \right)p\left(\varphi |\theta \right). \end{array}$$

we can proceed to maximize the likelihood of the data as before

$$\begin{array}{cc} \sum_{i=1}^{N}\log p\left(F\left(u^{i}\right)|\theta^{i}\right) & =\sum_{i=1}^{N}\log\sum_{\varphi }p\left(F\left(u^{i}\right)|\theta^{i},\varphi \right)p\left(\varphi |\theta^{i}\right), \end{array}$$

but in this case, Hypothesis 2 does not apply and so the expression does not simplify. If that is the case, we propose a generalization of Hypothesis 2 which may still hold.

##### Likelihood is Peaky in Different Subsets of Data

If we can split the *data* in subsets (we chose a two dataset partition for ease of presentation but the reasoning extends straightforwardly to an arbitrary number of sets.) $D_{1}={\left\{(u^{i},\theta^{i})\right\}}_{i=1\ldots N-P}$ and $D_{2}={\left\{(u^{i},\theta^{i})\right\}}_{i=N-P+1\ldots N}$, where the data were possibly reordered, such that for data in $D_{1}$ the likelihood $p(F\left(u^{i}\right)|\theta^{i},\varphi )$ is close to zero for all but single ${\tilde{\varphi }}_{1}$ and likewise for $D_{2}$, then we obtain the following upper bound for the log likelihood of observing the data:

$$\begin{array}{cc} \sum_{i=1}^{N}\log p\left(F\left(u^{i}\right)|\theta^{i}\right) & =\sum_{i=1}^{N}\log\sum_{\varphi }p\left(F\left(u^{i}\right)|\theta^{i},\varphi \right)p\left(\varphi |\theta^{i}\right)\\ \; & =\sum_{i=1}^{N-P}\log\sum_{\varphi }p\left(F\left(u^{i}\right)|\theta^{i},\varphi \right)p\left(\varphi |\theta^{i}\right)+\\ \; & \sum_{i=N-P+1}^{N}\log\sum_{\varphi }p\left(F\left(u^{i}\right)|\theta^{i},\varphi \right)p\left(\varphi |\theta^{i}\right)\\ \; & =\sum_{i=1}^{N-P}\log p\left(F\left(u^{i}\right)|\theta^{i},{\tilde{\varphi }}_{1}\right)p\left({\tilde{\varphi }}_{1}|\theta^{i}\right)+\\ \; & \sum_{i=N-P+1}^{N}\log p\left(F\left(u^{i}\right)|\theta^{i},{\tilde{\varphi }}_{2}\right)p\left({\tilde{\varphi }}_{2}|\theta^{i}\right). \end{array}$$

we thus have effectively two separate maximization problems, which we can proceed to solve in either of the likelihood maximization strategies presented above. The reasoning extends to an arbitrary number of subsets. We note that *i* indexes *observations*, hence that $\theta^{i}=\theta^{j}$ for i≠j. It could very well be that *every* $\theta^{j}$ is repeated. If that is the case, there are two concurrent physical processes, which we would discover by fitting each subset of the data.

##### Combining Separate Models

The method produces effectively two models for the same dataset. In order to recombine these models, assume for simplicity that every observed θ is in both $D_{1}$ and $D_{2}$, the same number of times. Then the likelihood is

$$\begin{array}{cc} \sum_{i=1}^{N}\log p\left(F\left(u^{i}\right)|\theta^{i}\right) & =\sum_{i=1}^{N/2}\log p\left(F\left(u^{i}\right)|\theta^{i},{\tilde{\varphi }}_{1}\right)p\left({\tilde{\varphi }}_{1}|\theta^{i}\right)+\sum_{i=N/2+1}^{N}\log p\left(F\left(u^{i}\right)|\theta^{i},{\tilde{\varphi }}_{2}\right)p\left({\tilde{\varphi }}_{2}|\theta^{i}\right)\\ \; & =\sum_{i=1}^{N/2}\log p\left(F\left(u^{i}\right)|\theta^{i},{\tilde{\varphi }}_{1}\right)+\sum_{i=N/2+1}^{N}\log p\left(F\left(u^{i}\right)|\theta^{i},{\tilde{\varphi }}_{2}\right)+\\ & \sum_{i=1}^{N/2}\log p\left({\tilde{\varphi }}_{1}|\theta^{i}\right)+\sum_{i=N/2+1}^{N}\log p\left({\tilde{\varphi }}_{2}|\theta^{i}\right). \end{array}$$

if we use the second method (maximizing a lower bound of the likelihood), we use the sum of the distance to the nearest neighbors of each datum as a measure of the likelihood of the data associated with each process.

#### 3.4.4. A Remark on Learning the Feature Space

As we noted in Section 3.3, learning the relationship between θ and the parameters of a differential equation is a considerable simplification. We do so by *choosing* a feature projection with the desired characteristics.

It is certainly possible to *learn* this feature projection, given enough data. To understand what is at stake, consider the setting of Section 3.4.3: we are to use the *same* number of training examples to maximize the likelihoods $\sum_{i=1}^{N}\log p\left(F\left(u^{i}\right)|\theta^{i},\tilde{\varphi }\right)$ and $\sum_{i=1}^{N}\log p\left(\tilde{\varphi }|\theta^{i}\right)$, where now *F* is to be learned.

To ground intuition, consider the problem of learning a linear mapping—the number of parameters of the linear mapping is a lower bound of the more general task. Consider the first task, that of learning the relationship between the parameters of the differential equation and the physical observables. Typically, the number of parameters of a differential equation $n_{\varphi }$ is of order zero, and the number of physical observables $n_{\theta }$ is of order one. The number of parameters to learn a linear mapping between these spaces $n_{\varphi }\times n_{\theta }$ is thus of order one. As for the second task, consider that physical fields are typically discretized *n*, and typically take values on a *m*-polytope, for a total number of features of $n^{m}$. In the task of predicting laser patterns, we have n=224 and m=2. The number of features is of order three, which brings the number of parameters of the linear mapping between the two spaces to order seven—seven orders of magnitude more than in the previous case.

If the number of training examples is large, the two tasks can be solved in the sense that a small bound on generalization error can be found. But if the number of training examples is small—which we can define in terms of providing an acceptable bound to generalization for the first task—then the second task, since the bound on true risk is given by an increasing function of complexity, cannot be acceptably solved with the same number of data.

## 4. Predicting Novel Laser Patterns with Few Data by Integrating Partial Physical Information

We now apply the framework described in the previous section to predicting new laser patterns. This problem is strictly in the scope of inverse problem theory, which aims at estimating physical model parameters based on observations, with the added difficulty of having few observations at a single moment in time and only a partial model of the physical process: the Swift-Hohenberg (SH) equation [28], a 4th-order partial differential equation on the plane which can be seen as a maximally symmetric model of convection.

In spite of Hypotheses 1 and 2 the problem remains severely ill-posed and biased since the SH prior is only an approximate model of the dynamics. Our general strategy to tackle this problem relies on finding a feature transformation *F* to remove some of this degeneracy.

We integrate the physical information in the SH equation in two ways: our first approach is based on training a Deep Neural Network surrogate of an SH solver [49,50] on the great number of solutions of the SH equation, in the image of *F*. To our best knowledge, this is an original approach. Neural Network surrogates have been shown to provide accurate solutions of Partial Differential Equations (PDE) solvers at a fixed computational cost and were applied successfully to notoriously difficult problems such as the three-body problem [51]. We then learn *h*, the mapping from laser parameters θ to SH parameters φ by backpropagating through the differentiable surrogate to minimize the mean squared error in feature space. Finally, we use the solver on the output of *h* in order to produce a novel pattern, given a set of laser parameters.

Our second strategy to integrate SH physical information is to label experimental data with the SH solver parameters of its nearest neighbor, in the image of *F*, amongst a great number of pre-generated solutions of the SH equation. This dramatically simplifies the problem of learning the relationship *h* between laser parameters θ and SH parameters φ, since φ is low-dimensional. As in the first case, we use the solver on the output of *h* in order to, given a set of laser parameters, produce a novel pattern.

Our experiments show that the second approach yields better results than the first, with good agreement between experimental and generated images (see Section 5.2). As we showed in Section 3, the second approach corresponds to maximizing a lower bound of the likelihood of the first, and the error that we incur can be controlled by the expected distance between pre-generated solutions of the SH equation in feature space.

### 4.1. The Physical Model

In this section, we present and motivate the Swift-Hohenberg equation as a partial physical model of laser-matter interaction leading to pattern formation, as well as the pseudospectral solver of the SH equation that we use to integrate this knowledge into the machine learning model, either via its generated solutions or via a neural network surrogate.

#### 4.1.1. The Swift-Hohenberg Equation as a Partial Model

The goal of this section is to introduce the Swift-Hohenberg equation, provide intuition on the pattern formation mechanism, and motivate it as a partial physical model.

##### The Swift-Hohenberg equation as a symmetric model of convection

The Swift Hohenberg equation was first presented in [28,52] in the context of Rayleigh-Bénard convection. It is a 4th-degree partial differential equation governing the time evolution of a certain real field u(x,t) (also called *instability*), by defining the relationship between its spatial and time derivatives. In one dimension, with N[u] representing some nonlinear functional of *u*, we have, in a dimensional form:

(10) $$\begin{array}{c} \frac{\partial u}{\partial t}=\left(\epsilon -1\right)u-2\frac{\partial^{2}u}{\partial x^{2}}-\frac{\partial^{4}u}{\partial x^{4}}+N\left[u\right] \end{array}$$

the term N[u] is a nonlinear term that controls the growth of the instability, the simplest choice being $N\left[u\right]=u^{3}$. With this choice, if *u* is a solution of the equation, then so is −u; and u=0 is a solution.

It can be seen by linear stability analysis that small perturbations of the u=0 solution will be amplified selectively: specifically, when ϵ>0, Fourier modes whose wave vector norm lies on a certain interval centered at 1, the width of which depends on ϵ, will be amplified, while all others will be attenuated. The left endpoint of the interval not being zero, this is a called a type I${}_{s}$ instability [1]. This type of selective attenuation leads to the formation of patterns by selecting perturbations with certain periodicities. Since the selection of wave vectors depends only on the norm, the patterns formed via this mechanism are isotropic.

**Figure 5:**
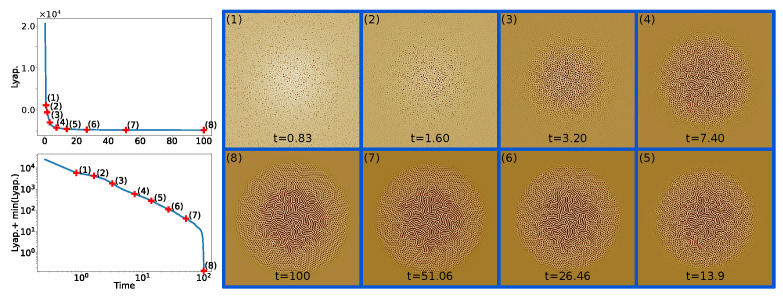
(**right**) SH solution with ϵ a centered 2D Gaussian ramp with a maximum of 0.8 and a standard deviation of 1/4 the domain size, to mimic the laser fluence distribution, and γ=−1.0, shown at several solver times *t*, with 1024² colocation points, represented as a 1024² heatmap (normalized to 1 since the SH eq. is adimensional); (**left**,**top**) SH Lyapunov functional $E\left[u\right]=\int_{\Omega }\frac{u}{2}\left(\nabla^{4}u+2\nabla^{2}u+u\right)+\frac{1}{4}u^{4}-\frac{\gamma }{3}u^{3}-\frac{\epsilon }{2}u^{2}dx$ (a functional of the field which has the property to decrease during the dynamics) with points corresponding to the solutions on the right highlighted. Note that, as expected, *E* converges asymptotically to a stable value; as can be observed from the field heatmaps on the right, as it does so, organized patterns form. (**left**,**bottom**) SH Lyapunov functional shifted up in log-log scale; SH solver terminates when the Lyapunov functional stops decreasing.

Although the SH equation was introduced as a model of a specific convection experiment, it can actually be motivated on general grounds, by appealing to symmetries. Specifically, given appropriate boundary conditions, the SH equation is the simplest equation with a type I${}_{s}$ instability that is isotropic, translation invariant, and invariance with respect to the u→−u substitution (see [1] for details). Crucially, the SH equation can be derived *from these assumptions alone* [1]. These symmetries are, in our case, verified, since cross-polarization was introduced specifically to make the patterns isotropic [17], which motivates our choice of SH as a partial model of pattern formation. The remarkable resemblance between real images on the one hand, and the images that we obtain by solving the SH equation on the plane using a finite-difference solver, and plotting the obtained solution as a heatmap, on the other, reinforces this choice (cf. Figure 5).

We break the symmetry with respect to u→−u by introducing a term $\gamma u^{2}$, the quadratic term allowing small amplitude destabilization and the existence of the hexagonal patterns which we observe experimentally, while the negative cubic term, which dominates for large amplitudes, still controlling the magnitude of the instabilities, which would otherwise grow without bound. The modified form of the Swift-Hohenberg equation used in this paper is thus

(11) $$\begin{array}{c} \frac{\partial u}{\partial t}=\left(\epsilon -1\right)u-2\frac{\partial^{2}u}{\partial x^{2}}-\frac{\partial^{4}u}{\partial x^{4}}+u^{3}-\gamma u^{2} \end{array}$$

##### The Swift-Hohenberg Equation: Intuition on Pattern Formation

**Figure 6:**
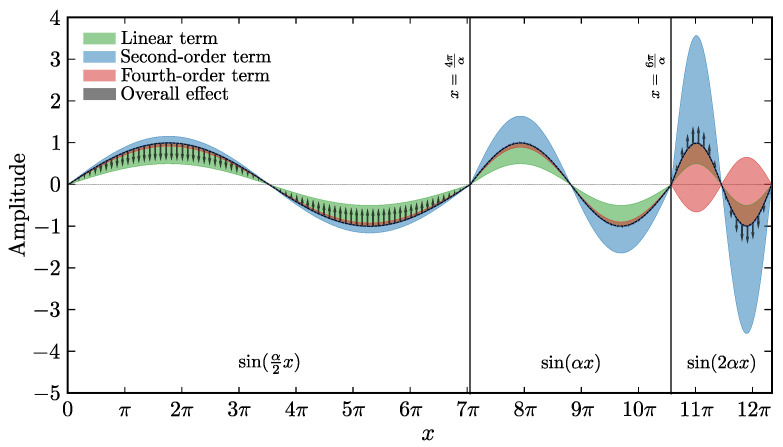
Action of the linear part of the one-dimensional Swift Hohenberg equation $\partial_{t}u=\epsilon u-{\left(1+\partial_{x}^{2}\right)}^{2}u+N\left[u\right]$ on three Fourier modes u=a(t)sin(kx). For small a(t), ignoring the nonlinear part and to first order in time, $\partial_{t}u=\left(\epsilon -1+2k^{2}-k^{4}\right)a\left(t\right)\sin\left(kx\right)$. The zeros of the factor on the right-hand-side are $k=\sqrt{1\pm \sqrt{1+\left(\epsilon -1\right)}}$. For ϵ=0.5 and $k_{1}=\sqrt{1-\sqrt{0.5}}$ the leftmost zero, the overall derivative of the mode $\sin(k_{1}x)$ is zero, as can be seen on the center plot. Modes with lower frequency will be attenuated, as can be seen on the leftmost plot, where we show the magnitude of the various terms on $\sin(0.5k_{1}x)$. Finally, the mode $\sin(2k_{1}x)$, for which the frequency lies between the zeros of the factor is amplified, as can be seen on the rightmost plot.

To provide intuition on how a simple equation motivated on symmetry grounds originates the complex patterns observed experimentally, we examine the relationship between the time derivative and each of the terms on the right-hand side *separately*. We shall see that it is the balance between the several terms of the SH equation which provides the complexity leading to the formation of patterns, as we illustrate in Figure 6.

Consider first $\frac{\partial u}{\partial t}=\left(\epsilon -1\right)u$. Since ϵ∈[0,1[, *u* will increase in time where u<0, and decrease where u>0, in proportion to |u|. If the field evolution were determined by this term alone, the amplitude of *u* for a perturbation of the zero solution would be everywhere attenuated with time.

As for the $\frac{\partial u}{\partial t}=-2\frac{\partial^{2}u}{\partial x^{2}}$ term, because of the negative sign, the value of *u* increases in regions that are convex (peaks) and decreases in regions that are concave (troughs). Determined by this term alone, perturbations of the zero solution would increase in magnitude, and more so where the frequency of *u* is high than where it is low.

The evolution under the fourth-order term is similar: the value of *u* decreases in time in regions where the fourth spacial derivative is positive and increases where it is negative. Fourth order derivatives are more difficult to visualize, so we examine its action on $\sin\left(q_{n}x\right)$, where $q_{n}$ are integer multiples of $\frac{2\pi }{L}$, since it is a well-known fact from Fourier analysis that any sufficiently well-behaved odd *f* function of period *L* can be written as weighted sum (superposition) of these functions (the Fourier modes).

Since the derivative can be taken term by term, we examine each $f_{n}$ individually. With $a_{n}\in R$ we have

$$\begin{array}{c} f\left(x\right)=\sum_{n}a_{n}\sin q_{n}x:=\sum_{n}a_{n}f_{n} \end{array}$$

the second derivative contribution of $f_{n}$ to the Swift Hohenberg dynamics is $2q_{n}^{2}f_{n}$: $f_{n}$ will increase in time where $f_{n}>0$ and decrease where $f_{n}<0$ (as discussed above), large wavelength features changing in amplitude faster than small wavelength ones. The fourth derivative term’s contribution is $-q_{n}^{4}f_{n}$: $f_{n}$ will decrease where $f_{n}>0$ and increase where $f_{n}<0$, proportionally to the inverse fourth power of its wavelength: large wavelength modes will see their amplitude change faster than small wavelengths.

To determine the overall effect, we sum up the contributions for each mode $\epsilon -1+2q_{n}^{2}-q_{n}^{4}$. The sign of this coefficient will determine if $f_{n}$ gets amplified (negative) or attenuated (positive). We conclude that modes will be amplified if $1+\sqrt{\epsilon }\leq q^{2}\leq 1+\sqrt{\epsilon }$, which determines the range of characteristic inverse wavelengths of the features in the observed patterns. We conclude by noting that this amplification is without bound, and that the nonlinear part N[u] plays an important role in controlling this growth.

##### The Swift-Hohenberg Equation Has Potential Dynamics

The Swift Hohenberg equation for field u(x,t) on a domain Ω with the periodic boundary conditions that are used in this paper has potential dynamics, meaning that there is a functional *E* of the field *u*, called the Lyapunov functional, such that

(12) $$\begin{array}{c} \dot{u}=-\frac{\delta E}{\delta u} \end{array}$$

where $\frac{\delta E}{\delta u}$ is the variational derivative of *E* with respect to variations δu. The functional *E* has the property of decreasing during the dynamics [1]. Since E[u] is also bounded below, it converges asymptotically to a stable value. The Lyapunov functional for the SH equation used throughout this paper is

(13) $$\begin{array}{cc} E\left[u\right] & =\int_{\Omega }\frac{u}{2}\left(\nabla^{4}u+2\nabla^{2}u+u\right)+\frac{1}{4}u^{4}-\frac{\gamma }{3}u^{3}-\frac{\epsilon }{2}u^{2}dx \end{array}$$

(14) $$\begin{array}{cc} \; & :=\int_{\Omega }E\left(u,\nabla^{2}u,\nabla^{4}u\right)dx \end{array}$$

as can be verified straightforwardly by calculating the functional derivative $\frac{\delta E}{\delta u}$ using the Euler-Lagrange equations

$$\begin{array}{c} \frac{\delta E}{\delta u}=\frac{\partial E}{\partial u}+\sum_{i=1,2}{\left(-1\right)}^{i}\nabla^{i}\cdot \frac{\partial E}{\partial \left(\nabla^{i}u\right)} \end{array}$$

the fact that *E* decreases during the dynamics will allow us to check for divergence by calculating it at fixed iteration intervals, as we explain in the following section.

#### 4.1.2. A Pseudo-Spectral Second Order Solver for the Sh Equation

In this section, we describe the finite-difference solver that we use in this paper. We shall also use this solver indirectly in the form of a surrogate neural network, which motivates some of the implementation choices that we describe below. We choose a second-order pseudo-spectral method, providing a good compromise between accuracy and speed. A pseudo-spectral method is a split-step method, a technique which we explain briefly below following mainly [53,54]. We use one spacial dimension in our discussion for ease of presentation, as it generalizes straightforwardly to the plane. We assume periodic boundary conditions throughout.

##### Operator Splitting

The SH equation can be written in terms of the sum of the action of a linear L and a non-linear N differential operator acting on *u*

(15) $$\begin{array}{c} \dot{u}=L\left[u\right]+N\left[u\right] \end{array}$$

with the action of L given by $L\left[u\right]=\left(\epsilon -{\left(1+\partial_{x}^{2}\right)}^{2}\right)u$ and the action of the nonlinear part defined as $N\left[u\right]=\gamma u^{2}-u^{3}$.

The idea is to discretize and integrate each of these parts in turn, which explains the name of the technique. Doing so allows us to solve the non-linear part in Fourier space, where its action reduces to multiplication, a considerable reduction in computational cost. The nonlinear part can be solved using a straightforward explicit method that is easy to implement. The solution $u(t_{n+1})$ of the equation above at a later time $t_{n+1}=t_{n}+dt$ can be obtained from that at $u(t_{n})$ via the exponential of the operator L+N [53]

$$\begin{array}{cc} u(t_{n+1}) & =\exp\left(dt(L+N)\right)u\left(t_{n}\right). \end{array}$$

integrating each part separately amounts to using the approximation

(16) $$\begin{array}{c} \exp\left(dt(L+N)\right)\approx \exp\left(dtL\right)\exp\left(dtN\right). \end{array}$$

the error that we incur in doing so can be established using the *Baker-Campbell-Hausdorff formula* (see [55] for a proof and [54] for applications to higher-order integrators), which states that for any non-commutative operators *X* and *Y*, the product of the two exponentials can be expressed in terms of the exponential of a single operator *Z*

$$\begin{array}{c} \exp(X)\exp(Y)=\exp(Z) \end{array}$$

where *Z* is given in terms of the commutators of *X* and *Y* in all except the linear term

(17) $$\begin{array}{c} Z=X+Y+\frac{1}{2}\left[X,Y\right]+\frac{1}{12}\left(\left[X,\left[X,Y\right]\right]+\left[Y,\left[Y,X\right]\right]\right)+\frac{1}{24}\left[X,\left[Y,\left[Y,X\right]\right]\right]\cdots \end{array}$$

Applying this formula with X=dtL and Y=dtN we conclude, since the commutator $\left[dtL,dtN\right]=dt^{2}\left[L,N\right]\neq 0$, that the approximation Equation (16) is order one—independently of the order of the methods that we choose for each of the individual parts. Specifically, we have

$$\begin{array}{cc} u(t_{n+1}) & =\exp\left(dtL+dtN\right)u(t_{n})\\ \; & =\exp\left(dtL\right)\exp\left(dtN\right)u\left(t_{n}\right)+O\left(dt\right). \end{array}$$

##### Second-Order Pseudo-Spectral Solver

It is possible to increase the order of approximation at the expense of extra intermediate steps [54]. In this paper, we use an order two splitting scheme, known as *Strang splitting* [56], which consists in taking a half step with the linear operator, a full step with the nonlinear operator, and a final half step with the linear operator (we examined a symmetric 3-fold Strang composition method [54] of order four, but the improvement was not sufficient for our purposes to justify the increase in execution time, which stems not only from the extra time stepping, but from the higher-order methods for each of the individual steps):

$$\begin{array}{cc} u(t_{n+1}) & =\exp\left(\frac{dt}{2}L\right)\exp\left(dtN\right)\exp\left(\frac{dt}{2}L\right)u\left(t_{n}\right)+O\left(dt^{2}\right). \end{array}$$

strang splitting increases the order of ordinary time-splitting to two provided we choose second-order methods to discretize each of the individual steps. We thus integrate the nonlinear part using Ralston’s method, an order two explicit Runge-Kutta method with the Butcher tableau (see e.g., [53] for notation)

$$\begin{array}{c} \begin{array}{ccc} 0 & 0 & 0\\ 2/3 & 2/3 & 0\\ & 1/4 & 3/4 \end{array} \end{array}$$

and integrate the linear part in Fourier space, where spatial derivatives amount to multiplication, using the trapezoidal rule, with Butcher tableau

$$\begin{array}{c} \begin{array}{ccc} 0 & 0 & 0\\ 1 & 1/2 & 1/2\\ & 1/2 & 1/2 \end{array}, \end{array}$$

the result then being transformed back to the original space. Using the Fast Fourier Transform [57] to calculate the discrete Fourier transform allows us to take this otherwise costly step at quasi-linear time complexity.

With $T(L,u^{t},k)$ and $R(N,u^{t},k)$ representing the Trapezoidal and Ralston’s numerical methods solving, respectively, $\dot{u}=L\left[u\right]$ and $\dot{u}=N\left[u\right]$ over a time step of *k* starting with data $u^{t}$, and denoting the discrete Fourier transform of a field *u* by *v*, our pseudospectral scheme reads

$$\begin{array}{cc} \; & u^{*}=F^{-1}\left\{T(L,v^{t},\frac{dt}{2})\right\}\\ \; & u^{**}=R\left(N,u^{*},dt\right)\\ \; & u^{t+1}=F^{-1}\left\{T(L,v^{**},\frac{dt}{2})\right\} \end{array}$$

where $u^{*},u^{**}$ are intermediate solutions.

##### Stability and Adaptive Time-Stepping

The Trapezoidal rule is an order two method with a region of absolute stability including the entire left half of the complex plane (which makes it suitable for the solution of stiff equations [53,58]); stability of the split step method is thus determined by that of the explicit step.

We use an adaptive time step of the order of the inverse of the spectral norm of the Jacobian of the nonlinear stepping operator to control the stability of the explicit scheme and improve the time performance of our solver. We control for divergence by examining the Lyapunov functional at fixed iteration intervals. As explained above, a growth of the Lyapunov functional implies divergence. When this happens, we go back to the time before Lyapunov functional growth, divide the time step by two, and proceed with the time stepping. This method is simple but allows us to automate the solver with minimum input on our part, which is important given the number of images that we need to generate.

#### 4.1.3. Training a Differentiable Surrogate of the Sh Solver

For our method, it is convenient to train a differentiable surrogate of the SH solver described above *in feature space*. Although strictly unnecessary, as we could alternatively use automatic differentiation, introducing a surrogate allows for a dramatic decrease in training time [50] for learning the relationship between the SH parameters and the laser parameters. Faster forward problem solutions provided by the surrogate will also prove convenient in evaluating feature choices by cross-validation.

**Figure 7:**
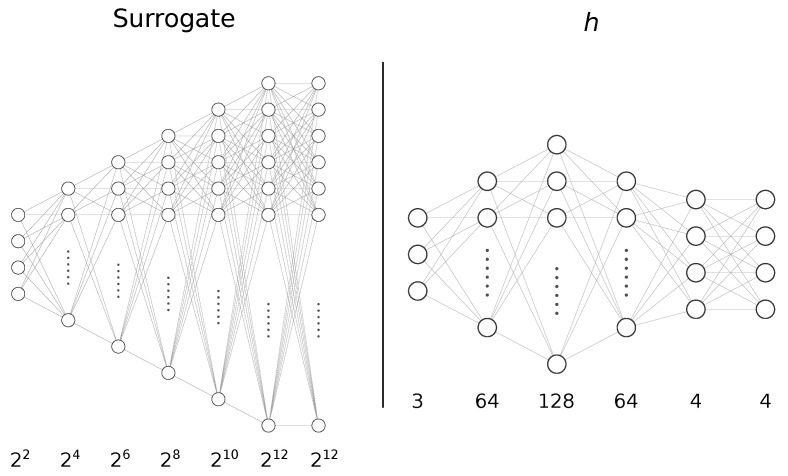
Neural network architectures: (**left**) differentiable SH surrogate, mapping from $\varphi \in R^{4}$ (Swift Hohenberg equation parameters, evolution time, and scale) to feature space ($R^{224\times 224}$). (**right**) mapping *h* from laser parameters $\theta \in R^{3}$ (laser Fluence, delay between pulses, and number of pulses) to $\varphi \in R^{4}$.

The surrogate consists of a neural network $f_{\omega }:\varphi \to F$ which learns the mapping between 4-dimensional φ (SH equation parameters, evolution time, and scale) to the projection of the SH solver-generated solutions in feature space, by minimizing the MSE. We use a 5 hidden-layer neural network with GeLU activations [59], which we initialize using He initialization [60] and regularize using weight decay. The number of units of the hidden layers are $2^{4},2^{6},2^{8},2^{10}$ and $2^{12}$. The architecture was chosen by cross-validation on a fraction of the data. The neural network was trained for 1000 epochs with early stopping (cf. Figure 7).

### 4.2. Choosing a Feature Space to Learn Patterns

We would like our framework presented in Section 3, which crucially depends on the existence of feature space in which the dependency of initial conditions is weak, to be generally applicable in the early stages of the experimental process, where there is neither enough physical knowledge to handcraft this invariance explicitly nor enough data to learn it directly. These two constraints motivate the use of features extracted by a large pre-trained model on a diverse dataset.

#### 4.2.1. Off-The-Shelf Feature Space Projector

**Figure 8:**
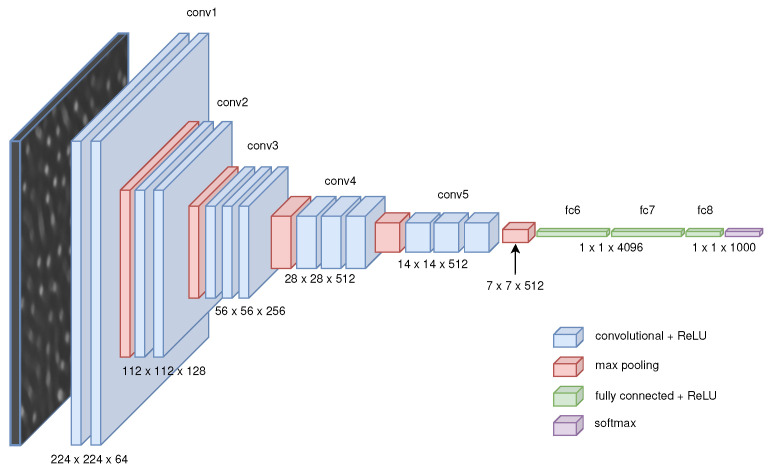
VGG16 network architecture. Our baseline features are extracted using layers “conv1” to “fc6” (inclusive).

We choose, as a feature projector, the first before the last layer of a pre-trained VGG16 [61], a deep convolutional network (CNN) model trained on ImageNet [62], a dataset of over 15 million labeled high-resolution images belonging to roughly 22,000 categories. See Figure 8 for details. Any complex model trained on a complex dataset like ImageNet is likely to acquire biases that depend on the dataset itself. Some of these may actually be good [63], but since they were not specifically controlled for, they could be undesirable for our task. In this sense, VGG16 appears to be a reasonable candidate in the context of our application.

Deep Convolutional Neural Networks achieves state-of-the-art performance on image classification tasks [64,65]. VGG16 in particular achieves 92.7% top-5 test accuracy on ImageNet, which is the main motivation for our choice, since the representation that a deep convolutional model needs to build (edges, textures, colors, and combinations thereof), in order to do well in such a classification task should be complex enough to represent the Swift-Hohenberg equation patterns as well. We further justify our choice by noting that this same feature extractor was used successfully to localize casting defects in (grayscale) X-ray images [66], and classify weld defects, both of which bear some similarities to our task.

#### 4.2.2. Learning Scale with the Features of a Scale-Invariant Network

There is still the problem of pattern scale, that needs to be learned. The success of convolutional neural networks and the scale-invariant nature of common image classification tasks led to extensive work in precisely the opposite direction: learning scale *invariant* features, rather than learning scale.

Convolutional neural networks such as VGG16 are not truly scale-invariant, though. Scale invariance is rather learned implicitly, even without a specific scale-invariant design [67], by training on datasets such as ImageNet, in which instances of the same class are represented at different scales. To obtain truly multi-scale scale invariant representations, we need to resort to local descriptors such as SIFT, which are popular in image processing [68], or recent deep learning techniques that focus specifically on scale invariance and equivariance [69,70,71,72], or impose it as a particular group symmetry [73].

Although learned implicitly, learned scale invariance for large convolutional models trained on ImageNet such as ResNet50 [65] and InceptionV3 [64] is quite good, as it was found recently that the probability of the correct class is approximately invariant to input size [74]. The authors show, however, that scale invariance varies with depth, information about the scale being chiefly present at intermediate layers, with invariance reached just before the softmax layer, and early layers focusing on local textures and small object parts. The authors then go on to show that by pruning the layers where the scale invariance is learned there are gains on a medical imaging task which, like our regression task, depends on scale.

Bearing this in mind, we prune the last two layers of a pre-trained VGG16, resulting in features encoding scale information and with enough complexity to represent the patterns of the Swift-Hohenberg equation. The resulting feature space has 4096 dimensions in the base case.

#### 4.2.3. Building the Datasets

We have two data types: one consisting of real SEM images, and the other of SH-generated images.

Because experimental manipulation is costly and time-consuming, the first dataset is small. It consists of 78 SEM images labeled with the laser parameter values $F_{p}$ peak *laser Fluence* (in J/cm²), *time delay* Δt (in $10^{-15}$s) and *number of pulses* *N*, of an area roughly 5 µm² size with a resolution of 237 pixels per µm.

The second dataset consists of a set of real fields generated by the SH equation with periodic boundary conditions on a square of side 224 according to the following procedure: we first sample uniformly the order parameter $\epsilon \in \left(0,1\right)$, the symmetry breaking parameter $\gamma \in \left[-2,2\right]$, and the system size $l\in \left(8,25\right)$ (in units of 2π); we seed the square with pointwise uniform initial conditions in $\left[0,\sqrt{\epsilon }\right]$ and evolve it according to the SH solver Section 4.1.2 until convergence, as assessed by the time derivative of the Lyapunov functional; we keep a maximum of ten snapshots of this evolution at regular solver time intervals *t*. Each of the resulting fields is labeled with the tuple ϵ,γ,l,t.

##### Pre-Processing

Since we shall ultimately compare real and generated images, we need to make a choice regarding image normalization. The experimental images are obtained via SEM microscopy, their intensity being preset. As for the generated images, the initial perturbation has a maximum amplitude of $\sqrt{\epsilon }$, and after a typical evolution time of $\frac{1}{\epsilon }$ [1], in the linear approximation, the maximum amplitude will remain a function of ϵ. To see this, note that the maximum growth rate $\sigma \left(q^{2}\right)=\epsilon -1+2q^{2}+q^{4}$ is at $q^{2}=1$, which corresponds to a maximum amplification at evolution time 1/ϵ of $e^{\epsilon /\epsilon }=e$, regardless of our chosen ϵ. Multiplying an ϵ-dependent maximum initial amplitude by a constant factor remains ϵ-dependent. The actual significance of such an amplitude depends also on the size of the domain, which is one of the parameters of the Swift-Hohenberg generated fields. Furthermore, since we take ten images at arbitrary time snapshots, the relationship between the maximum amplitude across fields is complex. This renders any normalization to our data other than image-wise difficult to establish (or essentially meaningless). This arbitrariness of levels is actually compatible with VGG16, in the sense that we expect that the ImageNet photographs that it was trained to classify were acquired with arbitrary levels and exposure.

We, therefore, normalize each image individually and subsequently transform the resulting array to pixel intensity space. Since VGG16 takes color images as its input, we use copies of our grayscale images as the input for the remaining two channels, before the final pre-processing of the input provided as a Keras [75] method (centering and normalizing each color channel with respect to ImageNet, and flipping RGB to BGR).

##### Subsampling

VGG16 takes 224 × 224-pixel images as inputs. In order to keep as much information as possible, we sub-sampled a maximum of ten such images at random orientations for each SEM image instead of downsampling the data. We allow for some variation in the number of samples due to the varying quality of SEM images, some of which have large patches consisting mostly of noise, which were removed.

##### Splitting the Dataset

**Figure 9:**
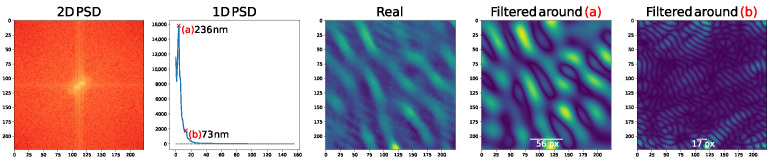
Pattern superposition: SEM image Fourier spectrum has two modal frequencies corresponding to two different patterns of different characteristic wavelengths. (**2D PSD**) is a heatmap of the logarithm of the Fourier power spectral density of the (**Real**) image. (**1D PSD**) is the fourth power (to exaggerate peaks) of the 1D PSD, obtained by azimuthally averaging 2D PSD along radii from the origin [76]. Peaks marked in red and labelled (a) and (b) are automatically extracted using a SimPy [77] method and correspond to the physical wavelengths displayed in the labels. The two images on the right are obtained from the real image by filtering out all wavelengths except the ones corresponding to the 1D PSD peaks, by multiplying it in Fourier space with a “Gaussian annulus” centered at the center of the image in Fourier space with a diameter equal to the wavelength of the peak. Image (**Filtered around (a)**) highlights the “top” pattern, whereas image (**Filtered around (b)**) highlights the “bottom” pattern.

As described in Section 3.4.3 one of the key assumptions in our framework is that the likelihood is peaky, possibly in different subsets of the data. We do observe that several SEM images have patches of two superimposed patterns of different length scales, an assertion that can be confirmed by analyzing their Fourier power spectrum, as shown in Figure 9.

**Figure 10:**
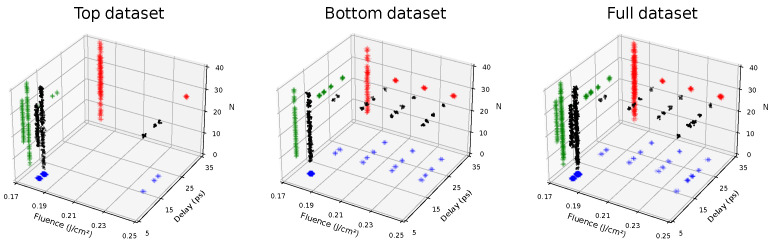
Real data laser parameters 3D scatter plots, with jitter for ease of visualization, for the “top”, “bottom”, and “full” datasets with, respectively 550, 435, and 985 data points. Data is represented in black, with projections on the Fluence/Delay plane in blue, Fluence/N plane in red, and Delay/N plane in green.

Since the SH equation is a single scale model, we built two expert-constructed datasets, “bottom” and “top”, with respectively 435 and 550 samples taken at random orientations, with a pattern that was found, in superimposed patterns, either bottom or on top. We concatenated the data into an “full” dataset consisting of 985 images. A visualization of the parameter ranges of each dataset can be seen in Figure 10.

#### 4.2.4. Selecting the Best Feature Space

In order to select the best feature space, we performed a KNN clustering task on the experimental images using Euclidean distance on each instance of the extracted features, and asked the domain experts to assess the quality of the extracted clusters, as described in Section 3.4.1.

We evaluate feature transformations consisting of variations of the features extracted by pruning the last two layers of a pre-trained VGG16: a version denoted “normalized” in which zero-variance features were removed and the remaining features scaled to unit variance; a version denoted “scale variant”, where we explicitly introduce scale information by concatenating the VGG16 extracted features with scale information consisting of a 1D power summary of the 2D power spectrum of each image obtained by azimuthally averaging the 2D power spectrum (PSD) along radii from the origin [76]; and finally, a version denoted “aligned”, in which a simple feature space alignment method [78] was used to align the distributions of the generated and real images.

Since normalization and alignment, as well as experts and MSE evaluations, are dataset-dependent, we compare each of these feature mappings in each dataset: “bottom”, “top”, “full”. We present a selection of the most relevant variations below.

##### Expert Clustering Results

**Figure 11:**
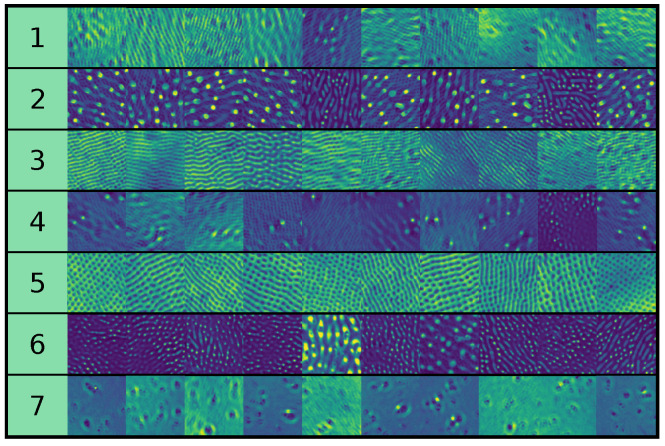
10 random samples for each of the 7 clusters (numbered 1 through 7 in the figure) obtained by k-means clustering (k = 7) for the f000 features, which consist of the full dataset projected into the VGG16 feature space without further modification.

As can be observed in Table 1 feature evaluation tends to favor the VGG16-extracted features without further transformation. We show cluster assignment for a random sample of images in the full dataset for the case of the VGG16-extracted features in Figure 11. Adding the PSD features generally reduces the expert-assessed clustering quality, which is consistent with the findings in [74]. If we choose to add the PSD features, however, we observe that the clustering quality improves by performing feature subspace alignment between real and SH-generated images, which can be explained by the fact that the PSD of real images contains small-scale information that is not present in SH-generated images, and that VGG16-extracted features are invariant to small-scale “noise”.

### 4.3. Learning h:θ→φ

Having access to an SH solver, the key task is learning the function *h* from laser parameter space to SH parameter space.

We begin by noting that the equation that we used throughout the paper is adimensional, derived on symmetry grounds only. For that reason, the *scale* is a hyperparameter of our solver, which also needs to be learned. The experimental setting described in [18], implies that there is a pattern solidification time that is independent of that of the process (which is chiefly controlled by the order parameter). This makes it unlikely that the observed patterns will be the long-time solutions to the SH equation: we are most likely observing transients. This is also apparent from inspection (e.g., top real image, third column in Figure 1. We thus choose as SH parameters the SH equation parameters ϵ and γ, the SH solver domain size *l* given in units of 2π, and the solver evolution time *t*. The laser parameters are $F_{p}$ the laser peak fluence in units of J/cm², the time delay between pulses Δt in picoseconds, and the number of laser pulses *N*.

We use the two methods described in Section 3.3 to learn *h*: directly Section 3.3.1 and by maximizing a lower bound Section 3.3.2.

#### 4.3.1. Learning *h* Directly

**Figure 12:**
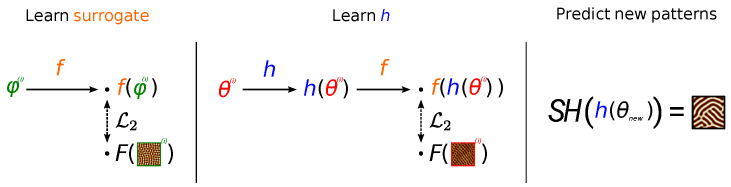
Learning *h* directly: (**left**) we begin training a surrogate *f* (orange) of the SH solver SH(·) in the image space of *F*, on a great number of pre-generated $\varphi^{\left(i\right)},F\left(SH\left(\varphi^{\left(i\right)}\right)\right)$ pairs (green), where $\varphi^{\left(i\right)}$ are SH parameters and $F(SH\left(\varphi^{\left(i\right)}\right))$ is the image in feature space of the solution generated by our solver with parameters $\varphi^{\left(i\right)}$, by minimizing the mean squared error in feature space; (**center**) we then learn h:θ→φ (blue) with real data ${\left\{\theta^{\left(i\right)},I^{\left(i\right)}\right\}}_{i=1\ldots M}$ (red) by minimizing the mean squared error in feature space; (**right**) finally, we generate patterns for unseen $\theta_{new}$ using the learned *h* and the solver: $SH(h\left(\theta_{new}\right))$.

In order to learn *h* directly, we use the pre-trained surrogate ${Solver}_{surr}:\varphi \to F$ described in Section 4.1.3 with frozen weights, with the following objective:

(18) $$\begin{array}{c} \min_{\alpha }\frac{1}{M}\sum_{i=1}^{M}{{∥Solver_{surr}\circ h_{\alpha }\left(\theta^{i}\right)-F\left(I^{i}\right)∥}_{2}}^{2} \end{array}$$

where $h_{\alpha }$ is a neural network parameterized by α, and M is the number of experimental data. We use a 4-hidden-layer neural network with GeLU activations, which we regularize using weight decay. The number of units of the hidden layers are 64, 128, 64, and 4 (cf. Figure 7). The network was trained over 1000 epochs with early stopping. We illustrate this procedure in Figure 12.

#### 4.3.2. Learning *h* Indirectly

**Figure 13:**
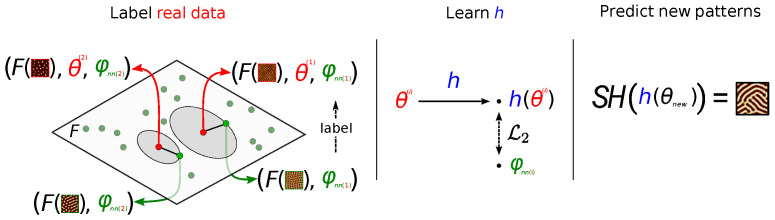
Learning *h* indirectly: (**left**) we begin by labelling each experimental datum *i* (red) with the SH parameter $\varphi_{nn(i)}$ of its nearest neighbor, in the image space of *F*, among a great number of pre-generated solutions of the SH equation (green); (**center**) we then learn h:θ→φ (blue) with data ${\left\{\theta^{\left(i\right)},\varphi_{nn(i)}\right\}}_{i=1\ldots M}$ by minimizing the mean squared error in parameter space; (**right**) finally, we generate patterns for unseen $\theta_{new}$ using the learned *h* and the solver: $SH(h\left(\theta_{new}\right))$.

In order to learn *h* indirectly, we first “train” a 1NN model with respect to the Euclidean distance on the pre-generated SH solutions $I^{i}$ with parameters in the set $\Phi_{pregen}$ (as described in Section 4.2.3) projected to feature space and assign to each experimental datum the SH parameters of its nearest neighbor. Explicitly:

(19) $$\begin{array}{c} \varphi^{i}=\underset{\varphi \in \Phi_{pregen}}{arg min}{∥Solver\left(\varphi \right)-F\left(I^{i}\right)∥}_{2} \end{array}$$

we then learn *h* by minimizing the mean squared error in feature space

(20) $$\begin{array}{c} \min_{\alpha }\frac{1}{M}\sum_{i=1}^{M}{∥\varphi^{i}-h_{\alpha }\left(\theta^{i}\right)∥}_{2}^{2} \end{array}$$

where $h_{\alpha }$ is a support vector regressor with RBF kernel, C=1.0 and ϵ=0.1, and *M* the number of experimental data. We illustrate this procedure in Figure 13.

## 5. Experimental Results

In this section, we present the experimental results. We begin by showing a comparison of cross-validation scores of the two methods in Section 4.3 as well as the feature mappings and datasets defined in Section 4.2.4. Cross-validation results in feature space show high variance, which is possibly a consequence of few training data, but could also be due to problems in training the surrogate, which is used to evaluate the scores (see the direct model discussion in Section 5.2 for details). In addition, the variety of feature transformations renders direct comparison between scores in different spaces difficult to interpret. As explained in Section 4.2.4, in our setting, the choice of feature space is best done by expert-evaluated cluster accuracy.

We then show a selection of predictions of the learned models and a map of parameter space for each dataset. We shall see that the choice of method is faced with much the same difficulties as the choice of feature mapping. We observe that better cross-validation scores do not necessarily lead to better predictions and that this is a consequence of the severe constraints of our problem.

We also observe that our best model is able to recover the main features (shape and scale) of test data and that the learned model is simpler for data where there is no pattern superposition. We show evidence that splitting the dataset improves the quality of the model, which suggests concurrent multiscale SH processes taking place, as explained in Section 3.4.3.

### 5.1. Cross Validation

Ideally, in order to compare the different feature mappings and methods, we would like to perform a 10-fold cross-validation of the mean squared error in feature space, which is closest to the task. The problem with this approach is that the image space of different feature mappings has different dimensions, which makes mean squared error comparison for different feature mappings meaningless. An alternative would be to normalize mean squared error by the variance, but this runs into the same problems that we discussed in Section 3.4.1 and yields inconsistent results. If we use this method to normalize the data the best feature space for the “full” dataset would underperform the expert-chosen features by 7%. In order to circumvent these difficulties, we can compare MSE in the 4-dimensional image of *h*, which we call *SH parameter space*. Although cross-validation scores become comparable across different feature spaces, this strategy is not without problems. Indeed, MSE in feature space is not necessarily a good measure of feature quality and the comparison across *methods* is unfair, since the indirect method relies on optimizing MSE in parameter space to with respect to the nearest neighbors, which is advantageous.

Baseline Method, Parameter Space

We define a baseline method as the “regressor” which predicts, for each datum, the SH parameters of its nearest neighbor in feature space. Instead of presenting the 10-fold cross-validation MSE results for this method, we actually present the leave-one-out cross-validation results, the latter being a lower bound of the former. The reason is expedience since the latter is simply the average squared distance between data in each dataset.

Cross Validation in Parameter Space

As we can observe in Table 2, cross-validation results for the direct method are compatible with the expert-based clustering results in Section 3.4.1, although the strength of this conclusion is limited by the high variance in the scores.

We also observe that cross-validation scores for the indirect method are best for features for which the PSD was appended and real and generated data features are aligned. Although the mean is strictly lower than in the case where the features are further normalized, the score ranges intersect.

A possible explanation for the better performance of the aligned feature choices is that the method crucially relies on the quality of the nearest neighbors. Fitting nearest neighbors in high dimensions is difficult since all points tend to be equidistant. Since aligned feature spaces are lower dimensional, their performance for this method should be better.

Baseline Method, Feature Space

We define a baseline method as follows: the “regressor” which predicts the features of the nearest neighbor in feature space. As in the case of the parameter space baseline above, we report the leave-one-out cross-validation MSE score, which is a lower bound of the 10-fold cross-validation score, consisting of the mean squared distance between data in each dataset.

Cross Validation in Feature Space

We present the 10-fold cross-validation scores in feature space for several choices of modified VGG16 features, datasets, and methods in Table 3. We note the high variance of the scores as well as the difference in scores across feature spaces spanning twelve orders of magnitude. We also note that the “direct” method is always better, as we evaluated it using the SH solver surrogate (doing a 10-fold cross-validation using the solver directly is impractical and would increase the variance even more), which makes for an unfair comparison with the 1NN “indirect” method.

### 5.2. Model Predictions

In this section we present the model-predicted SH parameters for the expert-selected features for unseen data from the “top”, “bottom” and “full” datasets, using the indirect method. We also present the direct method model-predicted SH parameters (for the full dataset only) which are inconsistent, and provide a possible explanation for this behavior.

#### 5.2.1. Indirect Method

**Figure 14:**
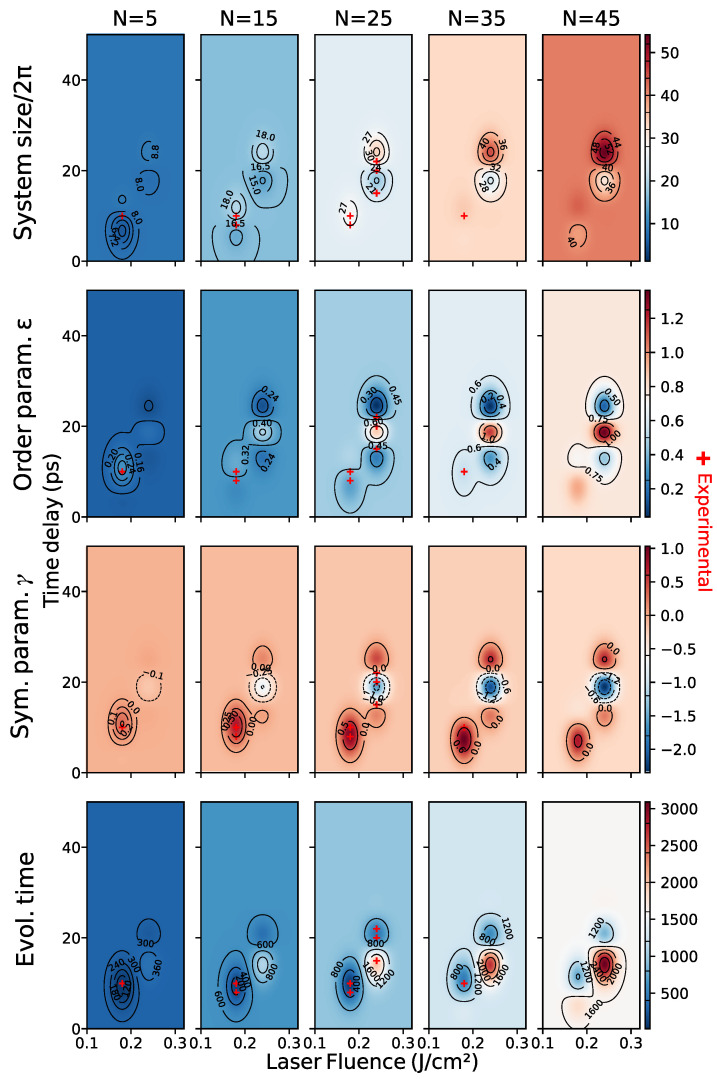
Each plot shows the predictions of the indirect model, trained on the “top” dataset, of a single SH parameter, as a heatmap (top to bottom: system size in multiples of 2π; order parameter ϵ; symmetry breaking parameter γ; solver evolution time) as a function of laser fluence, time delay, and number of pulses (respectively, x-axis and y-axis, and column). Experimental points are overlaid on each plot.

**Figure 15:**
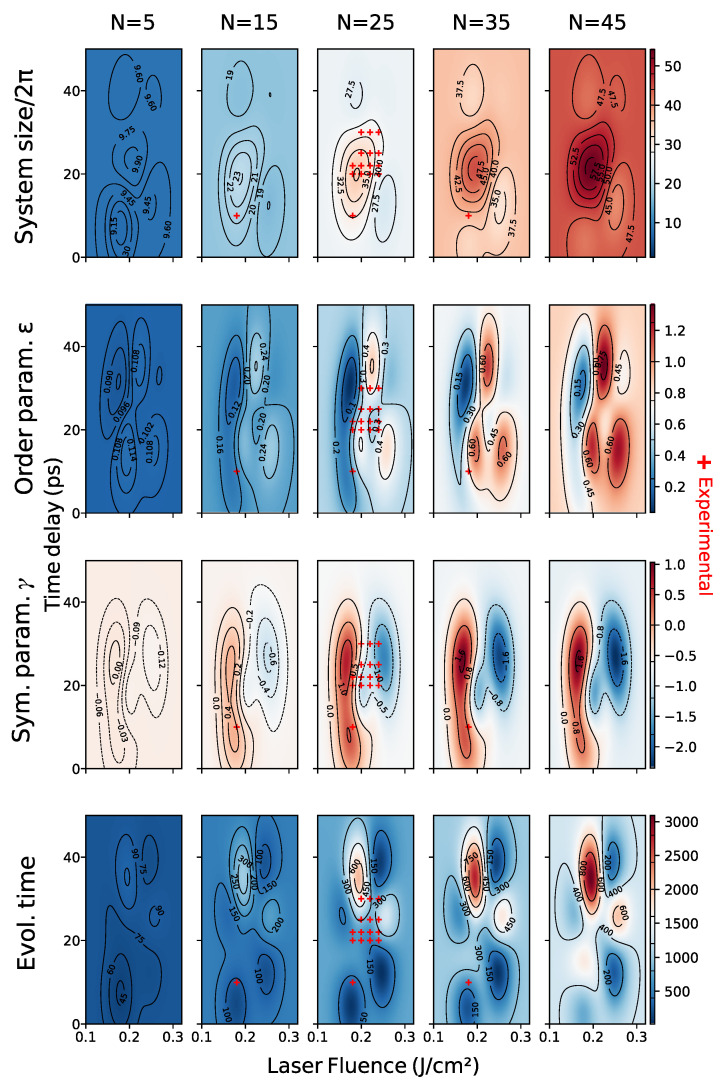
Each plot shows the predictions of the indirect model, trained on the “bottom” dataset, of a single SH parameter, as a heatmap (top to bottom: system size in multiples of 2π; order parameter ϵ; symmetry breaking parameter γ; solver evolution time) as a function of laser fluence, time delay, and number of pulses (respectively, x-axis and y-axis, and column). Experimental points are overlaid on each plot.

**Figure 16:**
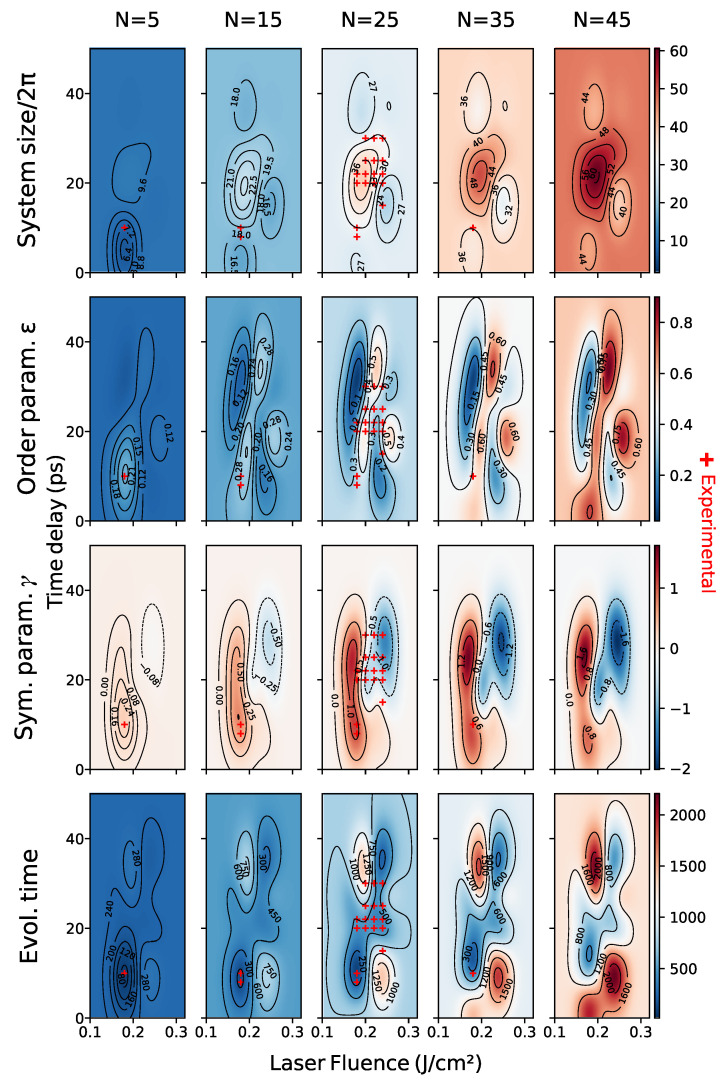
Each plot shows the predictions of the indirect model, trained on the full dataset, of a single SH parameter, as a heatmap (top to bottom: system size in multiples of 2π; order parameter ϵ; symmetry breaking parameter γ; solver evolution time) as a function of laser fluence, time delay, and number of pulses (respectively, x-axis and y-axis, and column). Experimental points are overlaid on each plot.

Comparing the model predictions for the “top”, “bottom”, and “full” datasets (cf. Figure 14, Figure 15 and Figure 16, respectively), we note that the first two are much simpler than the predictions learned on the combined dataset, which is consistent with the discussion about pattern superposition in Section 4.2.3 and the hypothesis that there is more than one SH process at play.

For all datasets, we note that the models struggle to extrapolate to regions for which there is no experimental data, in particular for laser fluence: predictions were generated for $F_{p}\in \left(0,0.5\right)$ for models trained on fluence data in (0.18,0.24); but as can be seen in the Figures above, we only show the fluence interval (0.1,0.32) because outside this range predictions are essentially constant. As for the time delay, experimental data Δt∈[8,25] but we manage to observe interesting prediction in the [0,50] range. For the laser parameter *N* (number of pulses), in spite of most experimental data being sampled at N=25, models manage to extrapolate to unseen *N* based on two sets of experimental series at ${\left\{(F_{p}=0.18\;J/{\mathrm{cm}}^{2},\Delta t=10\;\mathrm{ps},N)\right\}}_{N=6\ldots 36}$ and ${\left\{(F_{p}=0.18\;J/{\mathrm{cm}}^{2},\Delta t=8\;\mathrm{ps},N)\right\}}_{N=15\ldots 33}$

(the vertical lines in Figure 10). This opens up the possibility of improving predictions with sparse *additional* experimental data, with a focus on the $F_{p}$ laser parameter.

The SH parameter that shows the largest variation in predictions across values of *N* is the evolution time *t* parameter—which is what we would expect, since a larger number of pulses implies a process that is more extended in time. On the other hand, for all datasets, the complexity of the learned relationship increases with the number of observations, with sharp boundaries of rapidly varying parameters appearing where there is enough data.

**Figure 17:**
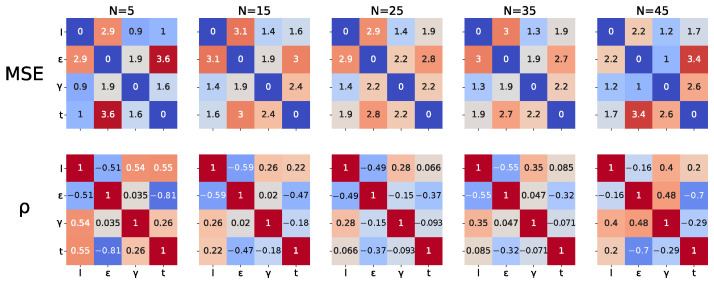
Mean squared error (**top row**) and Pearson’s coefficient (**bottom row**) between SH parameters l,ϵ,γ,t for indirect method predictions for the f000 features, for several values of *N* (columns).

Also noteworthy is the correlation between the various SH parameters l,γ,ϵ,t, as can be seen in Figure 17, which implies that one cannot design laser patterns freely. This correlation changes as the number of pulses *N* of the laser varies, which again testifies to the complexity of the underlying process.

##### System Size *l*

The parameter *l* is inversely correlated with the pattern characteristic size. Consistently with observations, *l* increases with *N*. Interestingly, this increase is not uniform: it is greater for large *l* regions in laser parameter space for low *N*, than for small *l* regions. The rapid transitions between *l* regions that can be observed for all values *N* are of possible interest to applications, in particular where other parameters are constant, as it would allow one to control the characteristic size of the particular pattern defined by the other SH parameters.

##### Symmetry Breaking Parameter γ

The SH parameter γ determines whether we observe holes or bumps. Of particular interest is the fact that for the “top” dataset, the transition from holes to bumps is in the Δt direction, whereas for the “bottom” dataset, the change is in the $F_{p}$ direction. This suggests that either two fundamentally different SH processes or a non-SH process are at play.

##### Order Parameter ϵ

To a larger parameter ϵ, farther from the onset, there correspond less ordered patterns, since there is a greater number of non-attenuated Fourier modes. The large ϵ low order patches at high fluence/low delay for the “bottom” dataset are consistent with this fact and match observations. For the “top” dataset, however, we observe an ordered pattern region of low ϵ and small *l* at low values of delay, which is more challenging to interpret. For the “full” dataset, the complexity of the ϵ isosurfaces in the central region of and around the isosurface of γ=0 is consistent with observations where a lower symmetry pattern is superimposed on a highly symmetric grid pattern (cf. Figure 1).

##### Evolution Time *t*

For constant l,ϵ,γ, symmetry increases with evolution time *t* as highly symmetric patterns require a large *t* to produce from a uniformly random state.

On the other hand, for all datasets, evolution time *t* tends to increase with *N*, which is consistent with the physical situation, as a large number of pulses increases the time the physical system is in a driven state. This increase, however, is not uniform across fluence, delay pairs: indeed we observe that the area of the region in laser parameter space of relatively long evolution time decreases with *N*.

##### Generating Novel Patterns

**Figure 18:**
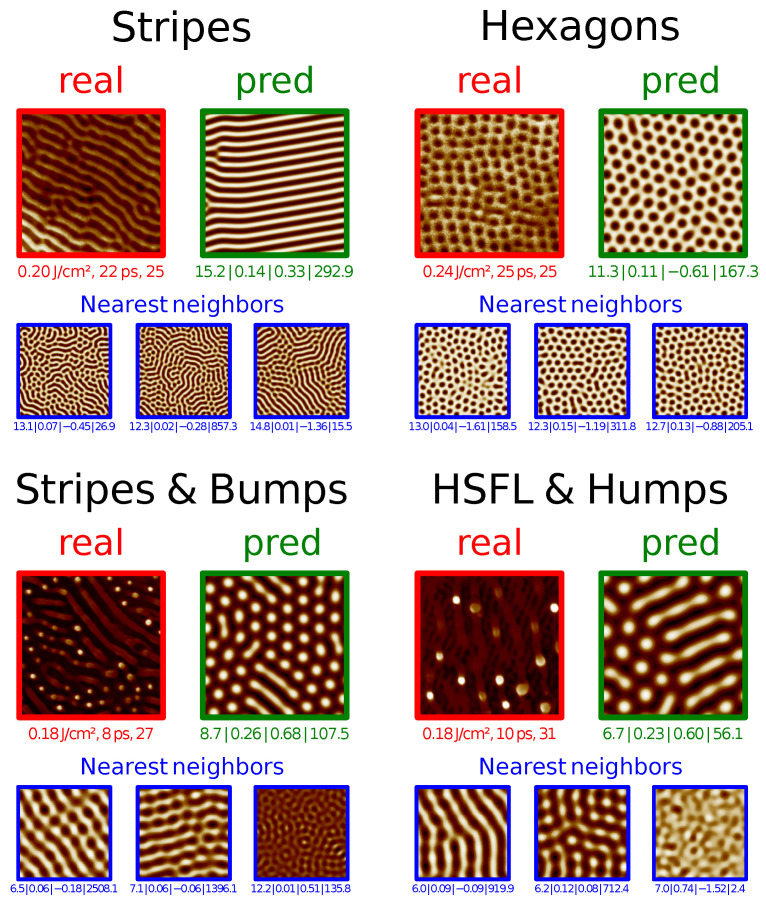
Each group shows experimental SEM images (**red**, never seen by the model), model predicted images (**green**, trained on the “full” dataset using the indirect method), given the same laser parameters and three nearest neighbors of the former among solver generated images (**blue**). Image labels, left to right: $F_{p},\Delta t,N$ (real images); *l*, ϵ, γ, $\tilde{t}$ (other images). All images are 224 by 224 pixels; for real images, 1 μm ≈ 237 pixels. Model’s predictions are better than the nearest neighbor since they integrate global information. On the “Stripes and Bumps” group, we observe nearest neighbors with different length scales, suggesting concurrent multi-scale SH processes taking place. Only one of these can be recovered by the ML model (the stripes, rather than the nanobumps), which integrates single-scale SH knowledge. On the “HSFL and Humps” group, the real image has a top, low-frequency pattern, and a finer grid pattern underneath. The model predicts the former, which is also the only among the nearest neighbors.

Instead of relying on the SH parameter interpretations above, one can use the SH solver to generate new patterns, as illustrated in Figure 13. We present the generated solutions from unseen laser parameters together with the corresponding real SEM image and nearest neighbors in feature space in Figure 18. Comparing solutions generated by the model from unseen test data to real SEM images allows further interpretation. The model does quite well for the ‘Stripes’ and ‘Hexagons’ group, as there is a single pattern to learn. For the two other groups, there is more than one pattern/feature superimposed on the SEM image. Note that the model does predict one of the observed patterns with the correct scale and shape but, by design, it cannot possibly predict the other. For the ‘Stripes and Bumps’ group, for instance, we observe nanopeaks and lower-frequency stripes among the nearest neighbors, whereas the model predicts the lower frequency pattern only. For the “HSFL and Humps” group, where the difference in pattern frequency is more pronounced, again the model focuses on the lower frequency pattern; the high-frequency pattern is no longer among the nearest neighbors.

#### 5.2.2. Direct Method

**Figure 19:**
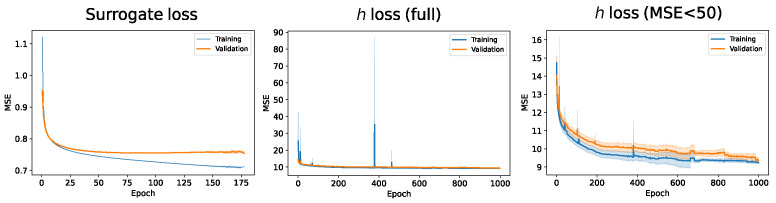
Direct method training and test (90/10) loss curves (mean and 95% confidence interval): (**left**) surrogate training on the large dataset of SH-generated data is stable, with narrow confidence interval bands; in all runs, early stopping was activated considerably earlier than the 1000 epoch limit (**center**) the h:θ→φ model, trained on few real data (the “full” dataset), on the other hand, is unstable; this behavior could be attributed to the dataset size, but the problem remains even for typical runs (**right**) detail of h:θ→φ training, with outliers for which training MSE exceeds 50 removed. A number of runs for which no early stopping was “activated” is evident, which provides further evidence of training instability.

While the surrogate trains well on SH-generated data, as can be seen in Figure 19, the *h* model, which trains on few real data, is unstable.

**Figure 20:**
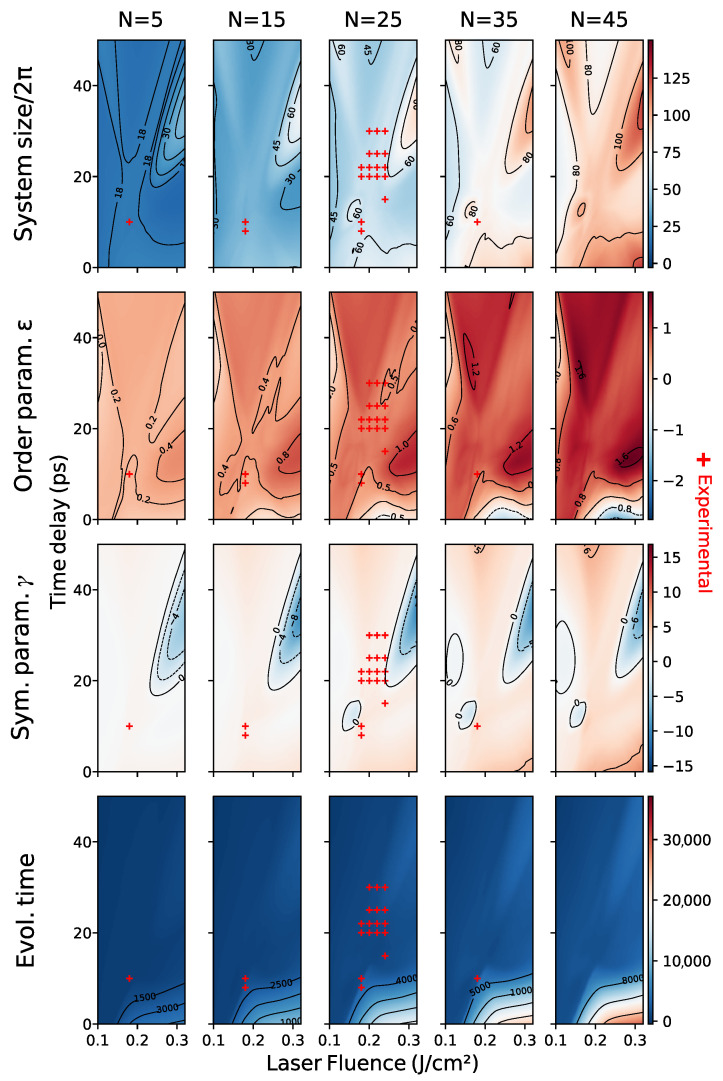
Each plot shows the predictions of the direct model, trained on the “full” dataset, of a single SH parameter, as a heatmap (top to bottom: system size in multiples of 2π; order parameter ϵ; symmetry breaking parameter γ; solver evolution time) as a function of laser fluence, time delay, and the number of pulses (respectively, x-axis and y-axis, and column). Experimental points are overlaid on each plot.

Although this small dataset artifact could partially explain why the direct method fails to yield accurate predictions, the problem remains even for “aligned” datasets and for typical training runs, closer to the center of the confidence interval, as can be seen in Figure 20.

It is known that surrogates using fully connected architectures often fail to achieve stable training by gradient descent and produce accurate predictions [79,80,81], and it is believed that this phenomenon is due to the difficulty of deep fully-connected networks in learning high-frequency functions [82]. We note that since we learn the surrogate on feature space, we cannot assess generated solutions’ accuracy directly. That being said, this unstable behavior is consistent with the very large variance observed in the cross-validation experiments in feature space Section 5.1.

While methods have been proposed to improve this behavior, either by introducing an adaptive learning rate [83] or by adjusting the weight of each term in the loss according to the singular values of the Neural Tangent Kernel [82], we do not use them in this paper, as the indirect method provides satisfactory results, is robust, and straightforward to implement.

## 6. Conclusions

In this paper, we integrated partial physical knowledge in the form of a PDE, the SH equation, to solve the problem of predicting novel nanoscale patterns in femtosecond irradiated surfaces. We showed that in the case of a self-organization process, the dual inverse problem of estimating state and equation parameters simplifies by choosing a feature transformation in the image space in which the initial conditions play a less important role. In the case where data is few and not time-series and the physical knowledge is only partial, this transformation can neither be learned nor derived: we use as transformation the higher-order features of a CNN pre-trained on a large dataset for a broad task. We proposed a principled approach to choosing such a feature transformation, and an expert-based quality measure of the features as well.

We integrated the PDE knowledge by implementing a fast and accurate second-order pseudospectral solver of the SH equation and then by using a great number of pre-generated solutions to learn a surrogate in feature space, on the one hand, and to label the few experimental data with SH parameters of the nearest neighbors in feature space, in the other. This technique allowed us to learn the relationship between laser parameters and SH parameters (with which novel laser patterns can be generated via the solver), a relationship that can be used as an experimental tool to guide new pattern discovery.

This led us to make a number of observations. First, in spite of the good agreement between the partial SH model predictions and experimental data, we also found evidence that there is more than one SH process at play. This leaves the door open to either using a generalized SH model that integrates several length scales, or exploring possibilities to combine multimodal single-scale data into a superimposed solution. Second, we observed that pattern features are not independent; finding novel patterns requires searching laser parameter space “creatively” by looking at regions where some SH parameter varies and some other does not. Third, we noted that although the model does not extrapolate well, it is still able to learn interesting features from little data. This opens the door to a dialectic approach to novel pattern discovery: one could simply acquire new experimental data iteratively in order to fill in the gaps in the laser parameter space until the predictions stabilize; since the SH model is already trained and data is pre-generated, integrating new experimental data only requires retraining a simple model on few data.

The main challenge in our approach is the scarcity of data. Although it is always possible, in principle, to acquire more experimental data, in practice the amount of data is unlikely to change because the cost is too high. In the laser pattern case, one possibility to circumvent this limitation is to combine data from experiments on several materials using domain adaptation [84], which would increase data by an order of magnitude and open the door to exploring patterns in unseen materials, which has great interest for applications.

## Figures and Tables

**Table 1:** Expert-assessed cluster quality for different feature choices. The best result for each dataset is in bold. Adding the PSD features does not improve the expert-assessed clustering quality. If we choose to add the PSD features, however, the clustering quality improves by performing feature subspace alignment between real and SH-generated images.

Dataset	PSD	Normalized	Aligned	Accuracy
full	False	False	False	**0.778**
full	False	False	True	0.776
full	True	True	True	0.699
full	True	True	False	0.671
full	True	False	True	0.634
bottom	False	False	False	**0.953**
bottom	False	False	True	0.952
bottom	True	True	True	0.812
bottom	True	True	False	0.712
bottom	True	False	True	0.793
top	False	False	False	0.857
top	False	False	True	**0.864**
top	True	True	True	0.855
top	True	True	False	0.770
top	True	False	True	0.709

**Table 2:** 10-fold cross-validation mean and standard deviation of MSE in SH parameter space for different methods (baseline, direct, 1NN), datasets, and feature spaces. Each row is labeled as: *dataset*, *Includes PSD*, *Normalized*, *Aligned*; 1 denotes True and 0 False. The b101 row, for example, lists the MSE and standard deviation in SH parameter space for the *bottom* dataset (b) where features include the PSD (1), are not normalized (0), and are aligned to with respect to the bottom dataset (1). The best scores for each method for each dataset are highlighted in bold. Note that the SH parameter space is the same for every row and column.

	Baseline	Direct	1NN
b000	8.019	**2.065 ± 0.629**	0.975 ± 0.202
b001	8.019	2.979 ± 1.146	0.870 ± 0.205
b101	8.019	3.701 ± 1.565	**0.745 ± 0.283**
b111	8.019	3.180 ± 2.739	0.765 ± 0.335
t000	8.015	**2.169 ± 0.976**	0.989 ± 0.150
t001	8.015	5.207 ± 5.226	0.914 ± 0.203
t101	8.015	5.323 ± 2.589	**0.609 ± 0.142**
t111	8.015	4.513 ± 6.354	0.615 ± 0.122
f000	8.008	2.351 ± 0.852	0.993 ± 0.950
f001	8.008	2.814 ± 1.814	0.912 ± 0.901
f101	8.008	3.693 ± 3.240	**0.659 ± 0.201**
f111	8.008	**2.006 ± 0.330**	0.694 ± 0.167

**Table 3:** 10-fold cross-validation mean and standard deviation of MSE in feature space for different methods (baseline, direct, 1NN), and feature spaces, for the full dataset. Each row is labelled as: *dataset*, *Includes PSD*, *Normalized*, *Aligned*; 1 denotes True and 0 False. The f000 row, for example, lists the MSE and standard deviation in SH parameter space for the *full* dataset (f) where features do not include the PSD (0), are not normalized (0) and are not aligned to with respect to the bottom dataset (0). Best scores across rows are highlighted in bold. Note that comparing across columns is meaningless, as MSE is calculated in different feature spaces.

	Baseline	Direct	1NN
b000	4.55 × 10^4^	**9.92 × 10^0^ ± 7.23 × 10^−1^**	1.46× 10^1^ ± 5.27 × 10^−1^
b001	3.70 × 10^4^	**8.93 × 10^1^ ± 9.73 × 10^0^**	1.37 × 10^2^ ± 7.7 × 10^0^
b101	2.75 × 10^9^	**3.95 × 10^6^ ± 1.44 × 10^6^**	7.99 × 10^9^ ± 1.45 × 10^9^
b111	2.17 × 10^−3^	**1.08 × 10^−4^ ± 2.00 × 10^−4^**	7.98 × 10^-3^ ± 1.21 × 10^−3^
t000	4.54 × 10^4^	**1.08 × 10^1^ ± 9.52 × 10^−1^**	1.43 × 10^1^ ± 8.04 × 10^−1^
t001	3.71 × 10^4^	**7.22 × 10^1^ ± 6.05 × 10^0^**	9.36 × 10^1^ ± 5.08 × 10^0^
t101	6.25 × 10^9^	**3.92 × 10^7^ ± 1.02 × 10^8^**	9.13 × 10^9^ ± 1.88 × 10^9^
t111	5.51 × 10^−3^	**1.07 × 10^−5^ ± 1.46 × 10^−6^**	1.25 × 10^−2^ ± 1.39 × 10^−3^
f000	4.73 × 10^4^	**1.08 × 10^1^ ± 1.13 × 10^0^**	1.43 × 10^1^ ± 2.73 × 10^−1^
f001	3.70 × 10^4^	**9.57 × 10^1^ ± 1.04 × 10^1^**	1.44 × 10^2^ ± 4.79 × 10^0^
f101	6.93 × 10^9^	**9.09 × 10^6^ ± 1.17 × 10^7^**	9.10 × 10^9^ ± 1.10 × 10^9^
f111	6.00 × 10^−3^	**1.38 × 10^−5^ ± 4.48 × 10^−6^**	1.02 × 10^−2^ ± 1.05 × 10^−3^

## Data Availability

The data presented in this study are available on request from the corresponding author, and will be made publicly available upon acceptance.
